# Food-derived dihydromyricetin and metabolic dysfunction-associated steatotic liver disease: a preclinical systematic review and meta-analysis

**DOI:** 10.3389/fnut.2026.1786758

**Published:** 2026-04-07

**Authors:** Dachuan Jin, Shunqin Jin, Tao Zhou, Guoping Sheng, Mingfei Yao, Peng Gao, Guangming Li

**Affiliations:** 1 Translational Medicine Research Center, Department of Liver Disease, The Sixth People’s Hospital of Zhengzhou, Zhengzhou, China; 2 Department of Radiology, Hebei Medical University, Shijiazhuang, China; 3 Department of Geriatric Medicine and Laboratory of Gerontology and Anti-Aging Research, Qilu Hospital of Shandong University, Jinan, Shandong, China; 4 Key Laboratory of Artificial Organs and Computational Medicine in Zhejiang Province, Department of Infectious Diseases, Shulan (Hangzhou) Hospital, Shulan International Medical College, Zhejiang Shuren University, Hangzhou, China; 5 State Key Laboratory for Diagnosis and Treatment of Infectious Diseases, National Clinical Research Center for Infectious Diseases, Collaborative Innovation Center for Diagnosis and Treatment of Infectious Diseases, The First Affiliated Hospital, College of Medicine, Zhejiang University, Hangzhou, China

**Keywords:** dihydromyricetin, hepatoprotective effects, lipid metabolism, meta-analysis, metabolic dysfunction-associated steatotic liver disease, oxidative stress

## Abstract

**Background:**

Dihydromyricetin (DHM) is a food-derived flavonoid widely investigated as a nutraceutical candidate for metabolic dysfunction–associated steatotic liver disease (MASLD) in preclinical models; however, its overall efficacy in diet-induced MASLD/NAFLD models has not been systematically quantified.

**Methods:**

This PRISMA 2020 systematic review and meta-analysis was registered in PROSPERO (CRD420251119087). PubMed, Embase, Web of Science Core Collection, the Cochrane Library, and four major Chinese databases were searched from inception to December 15, 2025. Controlled murine studies comparing DHM monotherapy with high-fat diet controls were included. Random-effects meta-analyses pooled standardized mean differences (SMDs) with 95% confidence intervals; risk of bias was assessed using SYRCLE’s tool.

**Results:**

Fourteen controlled studies were included. Compared with controls, DHM reduced hepatic triglycerides and total cholesterol, improved liver enzymes (ALT, AST, ALP), and decreased body weight and liver index. DHM improved serum lipid profiles (lower total cholesterol and LDL; higher HDL) and glucose homeostasis (lower fasting glucose and insulin). Antioxidant defenses increased (SOD, CAT, GSH, GSH-Px) with reduced malondialdehyde, while inflammatory markers (TNF-*α* and IL-6) decreased. At the signaling level, DHM increased the pAMPK/AMPK ratio. Heterogeneity was moderate to high for several outcomes, partly explained by dose and treatment duration.

**Conclusion:**

In murine diet-induced MASLD/NAFLD models, DHM shows promising multidomain benefits across various physiological outcomes, though some variability remains due to differences in study design. More standardized preclinical designs and well-controlled nutraceutical/clinical studies are needed to define clinically relevant, bioavailable dosing and efficacy.

**Systematic review registration:**

https://www.crd.york.ac.uk/PROSPERO/view/CRD420251119087, PROSPERO, Identifier CRD420251119087.

## 1 Introduction

Metabolic dysfunction-associated steatotic liver disease (MASLD), formerly known as non-alcoholic fatty liver disease (NAFLD), has become the most prevalent chronic liver condition worldwide, affecting approximately 25–38% of adults (1–11). As the hepatic manifestation of metabolic syndrome, MASLD is closely associated with central obesity, insulin resistance, type 2 diabetes mellitus, and dyslipidemia (12–16). Without timely intervention, the disease may progress to non-alcoholic steatohepatitis (NASH), advanced fibrosis, cirrhosis, or even hepatocellular carcinoma (17–19). Although the therapeutic landscape is rapidly evolving, currently approved pharmacotherapies target MASH with fibrosis in selected patient population, and there remains no widely accepted drug therapy for the broader MASLD spectrum (20–23). Lifestyle intervention and weight management remain foundational, but their efficacy is often limited by poor long-term adherence (24, 25).

Dihydromyricetin (DHM), a natural flavonoid primarily derived from *Ampelopsis grossedentata* (vine tea), has a long history of consumption in East Asia and has recently attracted increasing attention for its potential metabolic benefits (26–28). Preclinical studies suggest that DHM may alleviate hepatic steatosis, improve metabolic profiles, and attenuate oxidative stress in NAFLD models (29, 30). However, the evidence across individual studies remains inconsistent. For example, the reported effects of DHM on body weight and serum lipid levels vary substantially (30, 31): some studies observed increases in serum triglycerides after DHM administration, whereas others reported marked reductions (32, 33). Such discrepancies highlight the need for a systematic and quantitative synthesis to clarify the overall effects of DHM (34).

To date, no systematic review or meta-analysis has comprehensively evaluated the therapeutic efficacy of DHM in experimental NAFLD mouse models. To address this gap, we conducted a systematic review and preclinical meta-analysis of murine studies to integrate the existing evidence and provide a consolidated assessment of DHM’s hepatoprotective potential across multiple physiological domains relevant to MASLD pathogenesis. Given the growing interest in natural product-based interventions, our findings may also help guide the future development of DHM-derived pharmacological or nutraceutical formulations.

## 2 Materials and methods

### 2.1 Protocol and reporting standards

This systematic review and meta-analysis adhered to the PRISMA 2020 guidelines (Preferred Reporting Items for Systematic Reviews and Meta-Analyses) and the SYRCLE (Systematic Review Centre for Laboratory Animal Experimentation) recommendations for animal studies (35, 36). The study protocol was developed *a priori* and specified predefined eligibility criteria, data extraction procedures, and statistical methodologies. The protocol was prospectively registered in the International Prospective Register of Systematic Reviews (PROSPERO; registration number: CRD420251119087) and is publicly accessible at https://www.crd.york.ac.uk/PROSPERO/view/CRD420251119087.

### 2.2 Literature search strategy

A comprehensive literature search was conducted across eight databases from inception to December 15, 2025: PubMed, Embase, Web of Science Core Collection, and the Cochrane Library, as well as four major Chinese databases—China National Knowledge Infrastructure, Wanfang Data, VIP Database for Chinese Technical Periodicals, and Chinese Biomedical Literature Database. The search strategy incorporated both controlled vocabulary and free-text terms related to the intervention (dihydromyricetin) and the disease condition (MASLD, MAFLD, NAFLD, NASH). No language restrictions were applied. Restriction to murine studies was implemented during the screening stage based on predefined eligibility criteria.

Reference lists of eligible articles and relevant reviews were also screened to identify additional studies. Duplicate records were removed before screening. Two reviewers (S.J. And T.J.) independently screened titles and abstracts, followed by full texts, to determine study eligibility. Discrepancies were resolved through discussion or consultation with a third reviewer. The detailed PubMed search strategy is provided in Table 1 as an example with equivalent terms adapted to the indexing systems and syntax of the remaining databases.

**Table 1:** Search strategy on PubMed.

Search step	Search strategy
#1	“Dihydromyricetin”[Title/Abstract] OR “dihydro-myricetin”[Title/Abstract]
#2	“Non alcoholic fatty liver disease”[MeSH Terms]
#3	“Non alcoholic fatty liver disease”[Title/Abstract] OR “non alcoholic fatty liver disease”[Title/Abstract] OR “fatty liver nonalcoholic”[Title/Abstract] OR “liver nonalcoholic fatty”[Title/Abstract] OR “Nonalcoholic Fatty Liver”[Title/Abstract] OR “Nonalcoholic Fatty Livers”[Title/Abstract] OR “NAFLD”[Title/Abstract] OR “Nonalcoholic Fatty Liver Disease”[Title/Abstract] OR “Nonalcoholic Steatohepatitis”[Title/Abstract] OR “steatohepatitis nonalcoholic”[Title/Abstract] OR “MAFLD”[Title/Abstract] OR “metabolic associated fatty liver disease”[Title/Abstract] OR “MASLD”[Title/Abstract] OR “metabolic dysfunction-associated steatotic liver disease”[Title/Abstract] OR “metabolic dysfunction-associated fatty liver disease”[Title/Abstract] OR “NAFLD”[Title/Abstract] OR “NASH”[Title/Abstract] OR “MASH”[Title/Abstract] OR “metabolic associated steatohepatitis”[Title/Abstract] OR “steatosis of liver”[Title/Abstract] OR “steatohepatitis nonalcoholic”[Title/Abstract] OR “metabolic associated steatohepatitis”[Title/Abstract] OR “liver steatosis”[Title/Abstract]
#4	#2 OR #3
#5	#1 AND #4

Importantly, inclusion of four major Chinese databases ensured that relevant preclinical studies published in the Chinese-language journals were systematically captured. Because dihydromyricetin is derived from traditional Chinese medicine sources and much of the experimental work has been conducted in China, searching both international and Chinese databases strengthened the comprehensiveness of the review and reduced potential language and publication bias.

### 2.3 Eligibility criteria

Studies were included if they met the following criteria: (1) Animal model: Studies employing mouse models of NAFLD, regardless of strain or sex, in which the disease was induced by high-fat diet (HFD) or similar metabolic stressors. (2) Intervention: DHM monotherapy, administered at any dose, duration, or route, without co-treatment with other pharmacological or dietary agents. (3) Comparator: HFD-fed mice that did not receive DHM treatment. (4) Outcomes: Studies were eligible if they reported at least one outcome relevant to NAFLD-related pathophysiological processes. These included, but were not limited to indicators of general physiological status (e.g., body weight, liver index), lipid metabolism, liver function, glucose regulation, oxidative stress, inflammation, or hepatic signaling pathways. No restrictions were imposed on the type of outcome during the literature screening stage to ensure comprehensive capture of relevant evidence. Specific outcomes of interest were then determined based on the availability of data and included in the meta-analysis where appropriate. (5) Study design: Controlled animal experiments published as original research articles in peer-reviewed journals.

The exclusion criteria were as follows: (1) Studies based on *in vitro* experiments, human subjects, reviews, case reports, conference abstracts, or computational models; (2) Animal studies not conducted in mice (e.g., rat models); (3) Studies lacking a valid control HFD-fed control group or those using DHM in combination with other active treatments; (4) Duplicate publications or secondary analyses derived from the same dataset; (5) Full-text articles unavailable or missing essential quantitative data (e.g., standard deviation or sample size); (6) Absence of any prespecified outcome indicators.

### 2.4 Data extraction

Two independent reviewers screened the included studies and extracted data using a standardized data extraction template. Disagreements were resolved through discussion or, when necessary, adjudicated by a third investigator. The following information was collected from each eligible publication: (1) basic study information, including first author, publication year, and journal; (2) animal characteristics such as mouse strain, sex, age, and sample size; (3) NAFLD modeling methods (e.g., diet composition and induction duration); (4) intervention details, including DHM dosage, route of administration, and treatment duration; (5) control group characteristics (e.g., high-fat diet without DHM); and (6) outcome measures and corresponding numerical data.

When studies involved multiple DHM dose groups, only data from the highest therapeutically effective dose were extracted for the primary meta-analysis, to estimate the upper bound (maximum therapeutic potential) of DHM efficacy under the tested experimental conditions, consistent with prior preclinical meta-analyses (37, 38). The “highest therapeutically effective dose” used in the meta-analysis was based on the original studies included in this review. This dose was determined by the authors of each study based on their experimental design, where the highest dose tested was considered therapeutically effective in the respective models. This approach is commonly used in preclinical meta-analyses to avoid dilution of treatment effects by subtherapeutic doses, but may overestimate efficacy and was therefore interpreted conservatively in the Discussion (39). For studies reporting outcomes at multiple time points, results from the latest time point were preferentially selected. When relevant data were available only in graphical form, numerical values were estimated using GetData Graph Digitizer (version 2.26). When liver index was reported as a percentage, it was converted to a consistent metric prior to pooling (i.e., percentage values were divided by 100). If dispersion was reported as SE or SEM rather than SD, SD was calculated using SD = SE/SEM × √n. When dispersion was reported as mean ± S without specifying whether S represented SD or SEM, we conservatively treated S as SEM and converted it accordingly to avoid potential inflation of SMDs. All extracted data were entered into a pre-designed spreadsheet for subsequent statistical synthesis.

In this review, all extracted outcomes were categorized into eight functional domains to facilitate structured synthesis: (1) hepatic lipid profile; (2) liver enzymes; (3) anthropometric parameters; (4) serum lipid profile; (5) glucose metabolism; (6) oxidative stress; (7) inflammatory cytokines; (8) hepatic signaling proteins. Given the diversity of biochemical and molecular markers reported across animal studies, we did not pre-specify primary or secondary outcomes. Instead, all available endpoints were systematically extracted, and then grouped *post hoc* into eight functional domains to allow structured synthesis.

### 2.5 Risk of bias assessment

The risk of bias of all included studies was independently assessed by two reviewers using SYRCLE’s Risk of Bias (RoB) tool, which is specifically designed for preclinical animal research (40). This tool evaluates ten domains of bias, including sequence generation, baseline characteristics, allocation concealment, random housing, blinding of caregivers and investigators, random outcome assessment, blinding of outcome assessors, incomplete outcome data, selective outcome reporting, and other sources of bias. Each domain was rated as having a “low,” “high,” or “unclear” risk of bias based on the methodological details reported in each study.

Any discrepancies between the two reviewers were resolved through discussion, and if consensus could not be achieved, a third senior reviewer was consulted for arbitration. The overall risk-of-bias profile was visually summarized using Review Manager (RevMan, version 5.4).

### 2.6 Statistical analysis

All statistical analyses were performed using Stata version 15.1 (StataCorp, College Station, TX, USA). For continuous outcomes, standardized mean differences (SMDs) with corresponding 95% confidence intervals (CIs) were calculated to estimate the effects of DHM on NAFLD-related parameters. SMDs were used instead of weighted mean differences (WMDs) because the included studies assessed the same outcomes (e.g., triglyceride (TG), total cholesterol (Tche)) using different scales, units, or laboratory methods. Standardization enabled direct comparison across studies and improved the robustness of pooled estimates.

Given the considerable variability in animal models (e.g., age, strain, induction protocols) and experimental designs (e.g., dosage, intervention duration, outcome assessment methods), all pooled analyses were conducted using a random-effects model (DerSimonian and Laird method), irrespective of the results of heterogeneity tests. Between-study heterogeneity was assessed using the I^2^ statistic and Cochran’s Q test. Sensitivity analyses were performed using a leave-one-out approach for outcomes with at least three studies to evaluate the influence of individual studies on pooled estimates.

Publication bias was assessed using Egger’s test and visual inspection of funnel plots when ten or more studies were available for a given outcome (41). When publication bias was indicated, the trim-and-fill method was applied to examine the robustness of the pooled results (42). A two-tailed *p*-value < 0.05 was considered statistically significant. All analyses were reported in accordance with PRISMA 2020 guidelines to ensure methodological transparency and reproducibility. All analytical steps were independently cross-checked to ensure data integrity.

## 3 Results

### 3.1 Study selection

The systematic search retrieved 213 records from electronic databases and 1 additional record from other sources, yielding a total of 214 records. After removing 95 duplicates, 119 records (databases *n* = 118; other sources *n* = 1) underwent title and abstract screening, resulting in the exclusion of 41 records that were irrelevant to the research topic. The full texts of the remaining 78 articles (77 from databases and 1 from other sources) were assessed for eligibility.

**Figure 1:**
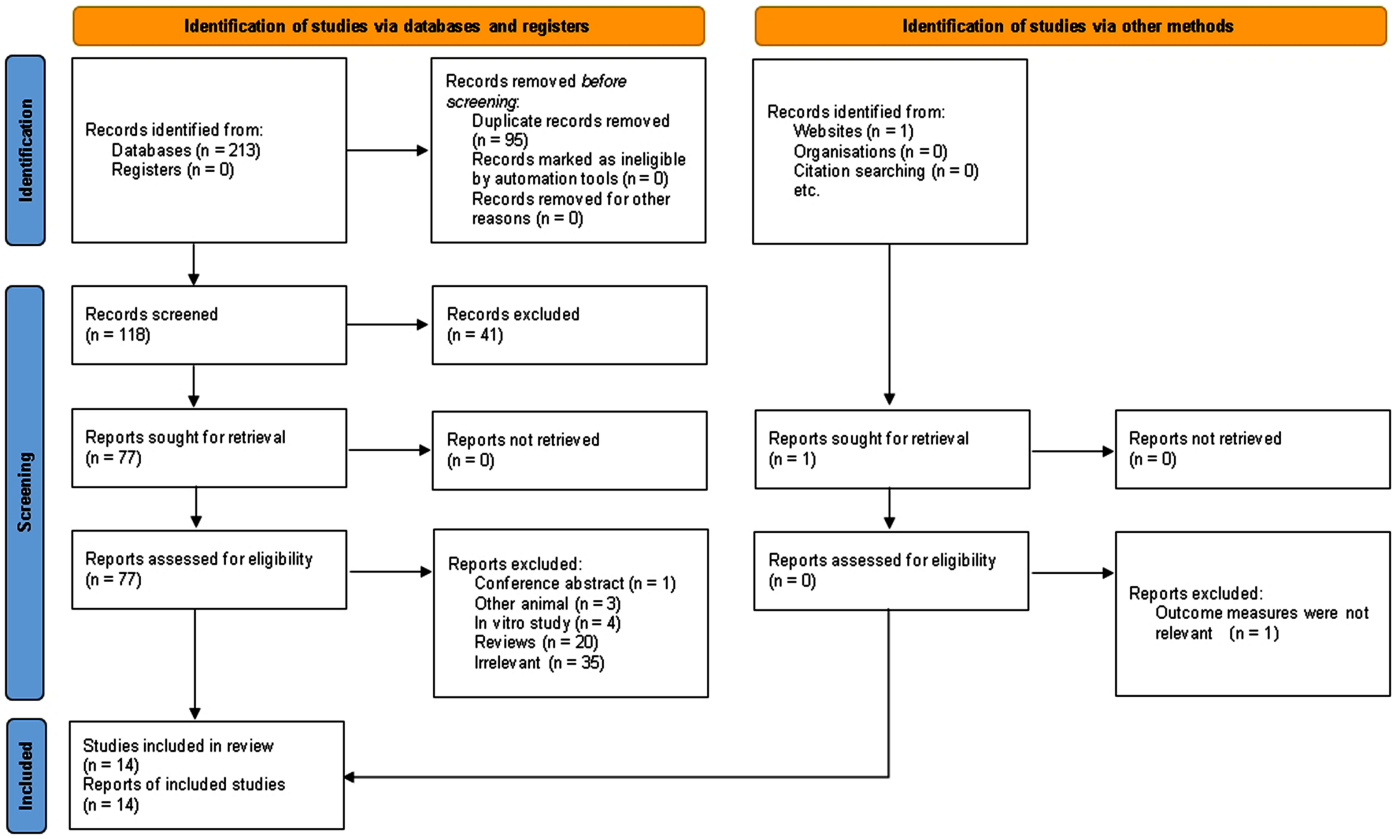
PRISMA flow diagram of study selection for this meta-analysis of DHM in MASLD animal models, showing database search, screening, eligibility assessment, and final inclusion. DHM, dihydromyricetin; MASLD, metabolic dysfunction-associated steatotic liver disease.

Of the 77 database-derived full-text articles, 63 were excluded for the following reasons: conference abstract (*n* = 1), non-murine animal studies (*n* = 3), *in vitro* studies (*n* = 4), reviews (*n* = 20), and studies irrelevant to the research question (*n* = 35). The single article identified through other sources was excluded after full-text review due to irrelevant outcome measures. Ultimately, fourteen murine NAFLD studies evaluating DHM interventions were included in the systematic review and meta-analysis (Figure 1).

All included studies were conducted in China between 2017 and 2024, employed high-fat diet–induced NAFLD models in male mice, and administered DHM orally either by gavage (25–1,000 mg/kg/day) or via drinking water at a concentration of 1.28 mg/mL for 4–16 weeks of DHM treatment. Key study characteristics are summarized in Table 1.

### 3.2 Characteristics of included studies

The fourteen included studies were published between 2017 and 2024, all conducted in China, and exclusively used HFD–induced NAFLD mouse models (29–33, 43–51). Most studies used wild-type C57BL/6 J mice, whereas two studies employed C57BL/6 J LDLR^−^/^−^ mice (44, 46) and another used C57BL/6 J ApoE^−^/^−^ mice (47) to model severe dyslipidemia. All studies were conducted in male animals. Detailed group sizes and total sample numbers for each study are provided in Supplementary Table S1.

DHM was administered as monotherapy in all included studies. Thirteen studies (29–32, 43–51) delivered DHM via oral gavage at doses ranging from 25 to 1,000 mg/kg/day, while one study (33) delivered DHM in drinking water at a concentration of 1.28 mg/mL. Because daily intake relative to body weight was not reported, the approximate daily dose was estimated at ~300 mg/kg/day using the formula *Dose = concentration × daily water intake ÷ body weight*, assuming a daily water intake of 4–6 mL and 18–20 g body weight for 7-week-old male C57BL/6 J mice (52–55). Treatment durations ranged from 4 to 16 weeks. All studies used vehicle-treated HFD-fed mice as controls (see Supplementary Table S1 for details).

The outcome measures reported across the included studies were diverse and were categorized into eight functional domains: (1) Hepatic lipid profile—triglycerides (TG) and total cholesterol (Tche). (2) Liver injury biomarkers—alanine aminotransferase (ALT), aspartate aminotransferase (AST), alkaline phosphatase (ALP). (3) Anthropometric parameters—body weight, liver index. (4) Serum lipid profile—TG, Tche, low-density lipoprotein cholesterol (LDL), and high-density lipoprotein cholesterol (HDL). (5) Glucose metabolism—fasting blood glucose and serum insulin. (6) Oxidative stress biomarkers—superoxide dismutase (SOD), catalase (CAT), glutathione (GSH), glutathione peroxidase (GSH-Px), and malondialdehyde (MDA). (7) Inflammatory cytokines—hepatic interleukin-1β (IL-1β), IL-6, and tumor necrosis factor-*α* (TNF-α), as well as serum TNF-α. (8) Hepatic signaling proteins—peroxisome proliferator–activated receptor-α (PPAR-α), carnitine palmitoyltransferase-1 (CPT-1), and the ratio of phosphorylated to total AMP-activated protein kinase (pAMPK/AMPK).

A summary of the study characteristics—including mouse strain, sample size, DHM dose, administration method, treatment duration, and outcome measures—is provided in Supplementary Table S1. As a whole, these studies contributed to a growing body of preclinical evidence supporting the hepatoprotective, anti-inflammatory, and antioxidative effects of DHM in diet-induced NAFLD. The consistent use of HFD-based murine models and the relatively uniform dosing regimens enhance comparability, while the inclusion of both wild-type and genetically susceptible strains permits broader exploration of DHM’s therapeutic relevance across varying degrees of metabolic dysfunction and disease severity.

### 3.3 Risk of bias, publication bias, and sensitivity analysis

The methodological quality of the 14 included studies was evaluated using SYRCLE’s risk-of-bias tool. None of the studies was rated as having a high risk of bias in any domain. For random sequence generation (selection bias), one study was judged as low risk, whereas the remaining studies were assessed as unclear due to insufficient methodological reporting. Regarding baseline characteristics, seven studies adequately reported comparable groups and were rated as low risk, while the others were judged as unclear. Allocation concealment was not described in any study and therefore all were classified as unclear risk. For random housing (performance bias), eight studies were considered low risk, and the remainder were unclear. Only one study explicitly reported blinding of investigators, and one reported random outcome assessment; all others were judged as unclear. Similarly, only one study reported blinding of outcome assessors, with the rest assessed as unclear. All studies were rated as low risk for incomplete outcome data, and selective reporting was generally well controlled, with only one study judged as unclear. No other sources of bias were identified. Overall, the methodological quality of the included animal studies was moderate. The primary concerns stemmed from inadequate reporting of randomization procedures, allocation concealment, and blinding, whereas outcome completeness and selective reporting were largely at low risk (Supplementary Figures S1, S2). These patterns indicate that incomplete reporting of key design safeguards remains a common limitation across the current preclinical evidence base.

Publication bias was assessed for six outcomes with ten or more studies: ALT, body weight, serum TG, serum Tche, serum LDL, and serum HDL (Supplementary Figure S3). Egger’s test indicated potential publication bias for serum Tche (*p* = 0.01), while no significant bias was detected for the remaining outcomes (all *p* > 0.05) (Supplementary Table S2). To further explore robustness, a trim-and-fill analysis was conducted for serum Tche; however, no trimming was performed, and the pooled effect size remained unchanged. Although Egger’s test did not indicate significant bias for serum TG (p > 0.05), the funnel plot appeared asymmetric; trim-and-fill analysis was therefore performed, which again showed no trimming and no change in the pooled estimate. This pattern may reflect the limited power of Egger’s test with a moderate number of studies and suggests that the observed asymmetry is more likely attributable to between-study heterogeneity rather than true publication bias.

Sensitivity analyses were conducted for 19 outcomes with at least three studies included. Leave-one-out analyses supported that sequential exclusion of any single study did not substantially alter the pooled estimates, supporting the robustness of the findings (Supplementary Figures S4–S11; inflammatory cytokines are shown in Supplementary Figure S10). Outcomes with fewer than three studies were not subjected to sensitivity analysis, as exclusion of one study would cause the results to be determined entirely by a single dataset, rendering such analyses uninformative.

Of all the above-mentioned outcomes assessed, 12 outcomes were associated with the study where the DHM dose was estimated based on assumptions about daily water intake (~300 mg/kg/day). These outcomes included body weight, serum TG, ALT, AST, hepatic MDA, SOD, GSH, GSH-Px, CAT, TNF-α, IL-6, and IL-1β. The sensitivity analysis showed that the estimated dose did not significantly affect the overall findings for these outcomes, indicating that the uncertainty from the estimated dose had minimal impact on the pooled results. While such uncertainties exist, their effect on the meta-analysis was negligible. Future studies would benefit from using more precise dosing methods to reduce such uncertainties.

### 3.4 Effects of intervention

To better reflect MASLD disease progression, the pooled outcomes are presented in a pathophysiologically ordered framework, moving from metabolic and lipid disturbances to organ injury, oxidative stress, inflammation, and upstream signaling pathways.

#### 3.4.1 Lipid profile

**Figure 2:**
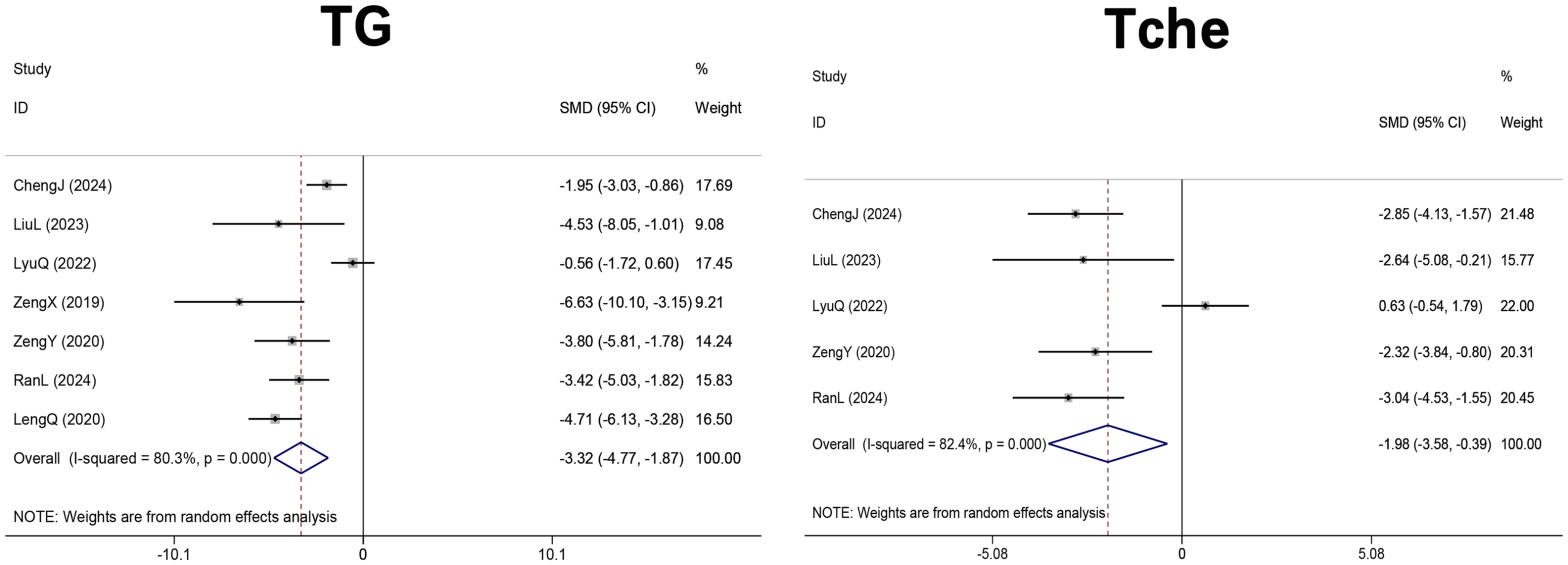
Forest plot of the effect of DHM versus control on hepatic lipid profiles in MASLD animal models. Pooled effect sizes are expressed as SMDs with 95% CIs using a random-effects model. DHM, dihydromyricetin; TG, triglyceride; Tche, total cholesterol; MASLD, metabolic dysfunction-associated steatotic liver disease; SMD, standardized mean difference; 95% CI, confidence interval.

Seven studies (29, 31, 33, 44, 46, 48, 51) (*n* = 106 mice) evaluated hepatic TG, and five studies (31, 33, 44, 46, 48) (*n* = 66 mice) assessed hepatic total cholesterol following DHM treatment in high-fat diet-induced NAFLD mice. Meta-analysis using a random-effects model supported a significant reduction in hepatic TG levels (SMD = −3.32, 95% CI: −4.77, −1.87, *p* < 0.001; I^2^ = 80.3%, *p* < 0.001) and in hepatic Tche (SMD = −1.98, 95% CI: −3.58, −0.39, *p* = 0.015; I^2^ = 82.4%, *p* < 0.001) in DHM-treated groups compared with controls (Figure 2).

**Figure 3:**
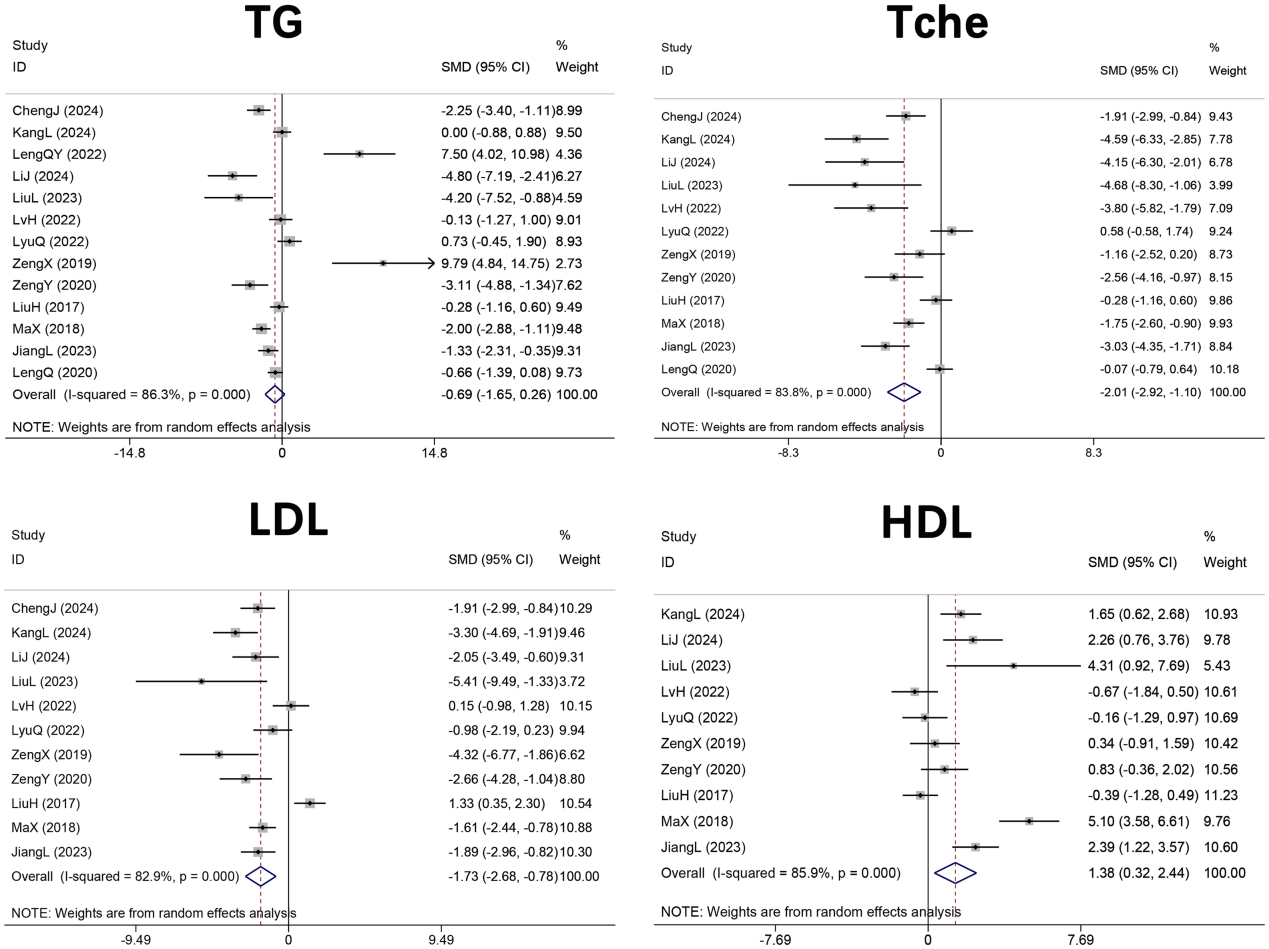
Forest plot of the effect of DHM versus control on serum lipid profiles in MASLD animal models pooled SMDs with 95% CIs were calculated using random-effects models. MASLD, metabolic dysfunction-associated steatotic liver disease; SMD, standardized mean difference; 95%CI, confidence interval; TG, triglyceride; Tche, total cholesterol; LDL, low density lipoprotein cholesterol; HDL, high density lipoprotein cholesterol.

Thirteen studies (29–33, 43–47, 49–51) (*n* = 216 mice) evaluated serum triglycerides, twelve (29–31, 33, 43–47, 49–51) (*n* = 204) assessed Tche, eleven (29–31, 33, 43–47, 49, 50) (*n* = 174) examined LDL, and ten (29–31, 43–47, 49, 50) (*n* = 154) measured HDL in HFD-induced NAFLD mice treated with DHM. Meta-analyses showed that DHM had no significant effect on serum TG levels (SMD = −0.69, 95% CI: −1.65 to 0.26; I^2^ = 86.3%, *p* < 0.001). In contrast, significant reductions were observed in serum Tche (SMD = −2.01, 95% CI: −2.92 to −1.10, *p* < 0.001; I^2^ = 83.8%, p < 0.001) and LDL (SMD = −1.73, 95% CI: −2.60 to −0.78, *p* < 0.001; I^2^ = 82.9%, p < 0.001), accompanied by a significant increase in HDL (SMD = 1.38, 95% CI: 0.32 to 2.44, *p* = 0.011; I^2^ = 85.9%, *p* < 0.001). Collectively, these findings indicate that DHM favorably modulates circulating lipid profiles by lowering Tche and LDL while elevating HDL levels (Figure 3).

#### 3.4.2 Anthropometric parameters

**Figure 4:**
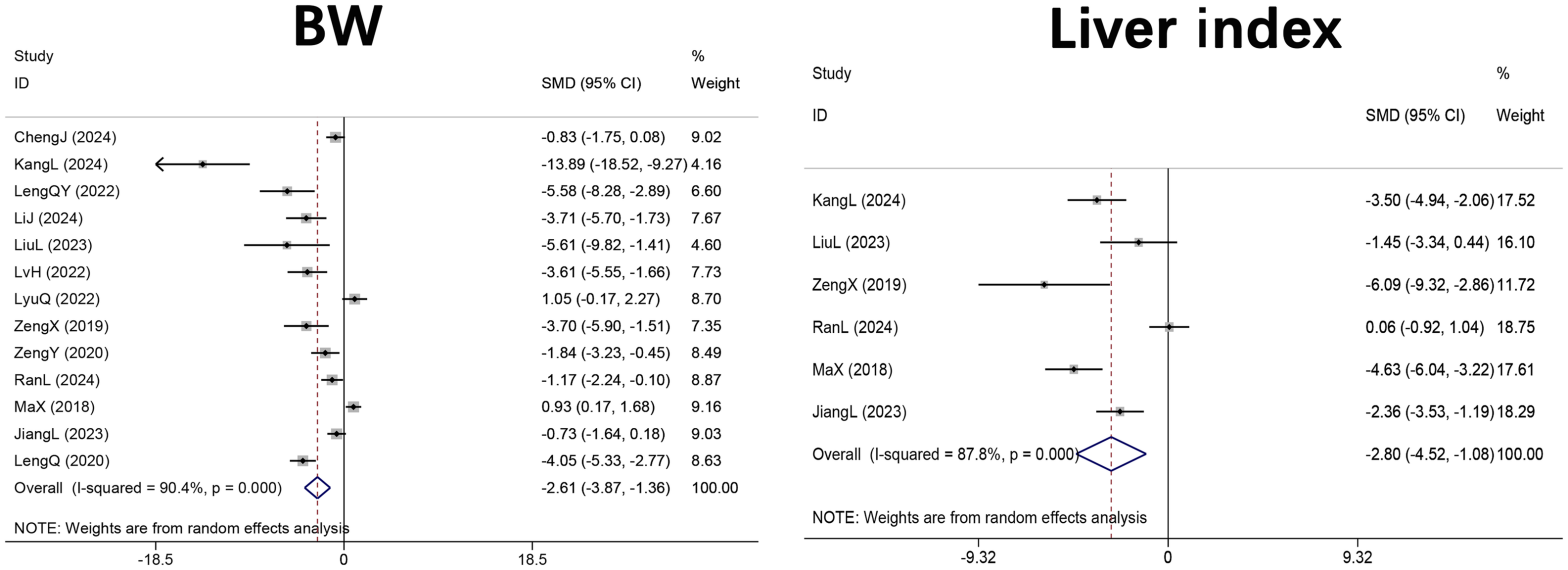
Forest plot of the effect of DHM versus control on anthropometric parameters in MASLD animal models. Effect sizes are presented as SMDs with 95% CIs using random-effects models. MASLD, metabolic dysfunction-associated steatotic liver disease; SMD, standardized mean difference; 95% CI, confidence interval; BW, body weight.

Thirteen studies (29–33, 43–46, 48–51) (*n* = 212 mice) examined the effects of DHM on body weight, and six studies (29, 30, 44, 48–50) (*n* = 102 mice) evaluated the liver index, defined as the ratio of liver weight to body weight, in HFD-induced NAFLD mice. The pooled analyses revealed significant reductions in both body weight (SMD = −2.61, 95% CI: −3.87 to −1.36, *p* < 0.001; I^2^ = 90.4%, *p* < 0.001) and liver index (SMD = −2.80, 95% CI: −4.52 to −1.08, *p* = 0.001; I^2^ = 87.8%, *p* < 0.001) in DHM-treated groups compared with controls (Figure 4).

#### 3.4.3 Glucose metabolism

**Figure 5:**
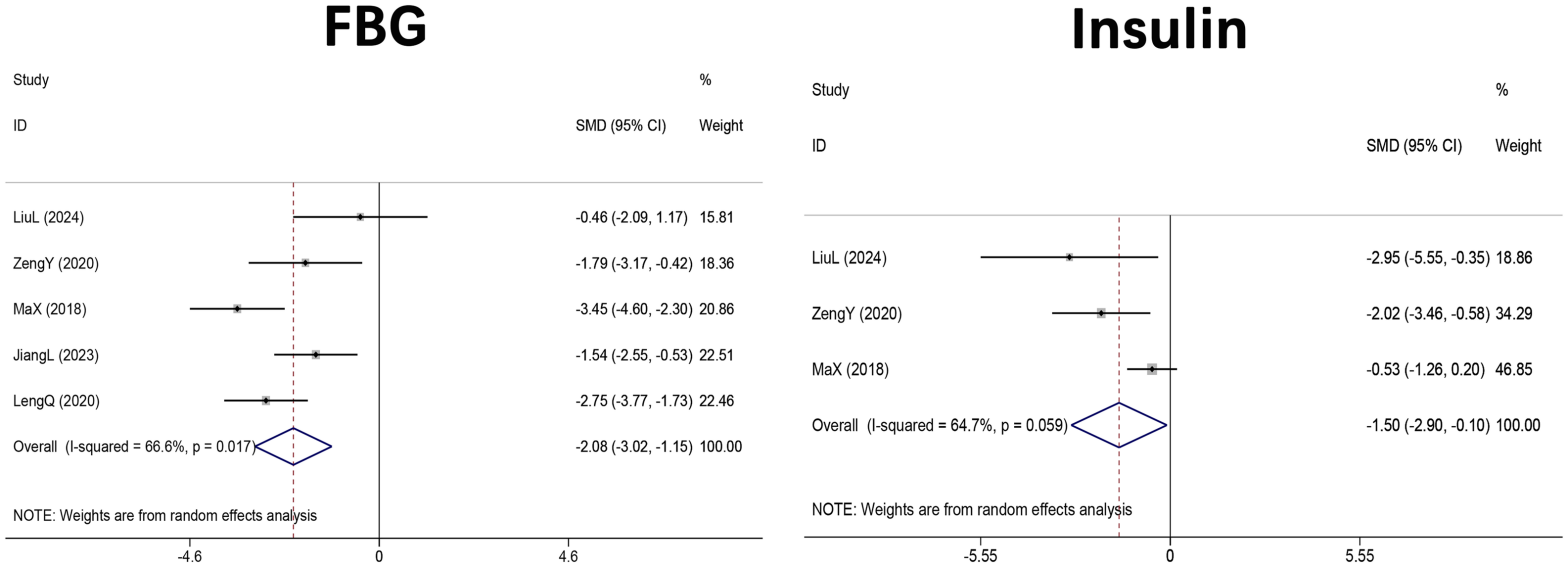
Forest plot of the effect of DHM versus control on glucose metabolism outcomes in MASLD animal models. Effect sizes are expressed as SMDs with 95% CI using random-effects models. MASLD, metabolic dysfunction-associated steatotic liver disease; SMD, standardized mean difference; 95% CI, confidence interval; FBG, fasting blood glucose.

Five studies (46, 49–51, 56) (*n* = 108 mice) evaluated FBG, and three studies (44, 46, 49) (*n* = 58) assessed fasting insulin concentrations in HFD-induced NAFLD mice. Meta-analysis supported a significant reduction in FBG following DHM administration compared with controls (SMD = −1.90, 95% CI: −3.09, −0.72, *p* < 0.001; I^2^ = 83.0%, *p* < 0.001). Similarly, DHM supplementation significantly reduced circulating insulin levels (SMD = −1.73, 95% CI: −3.27 to −0.19, *p* = 0.027; I^2^ = 79.9%, *p* = 0.007). These results suggest that DHM ameliorates glucose dysregulation and improves insulin sensitivity in murine NAFLD models (Figure 5).

#### 3.4.4 Liver injury

**Figure 6:**
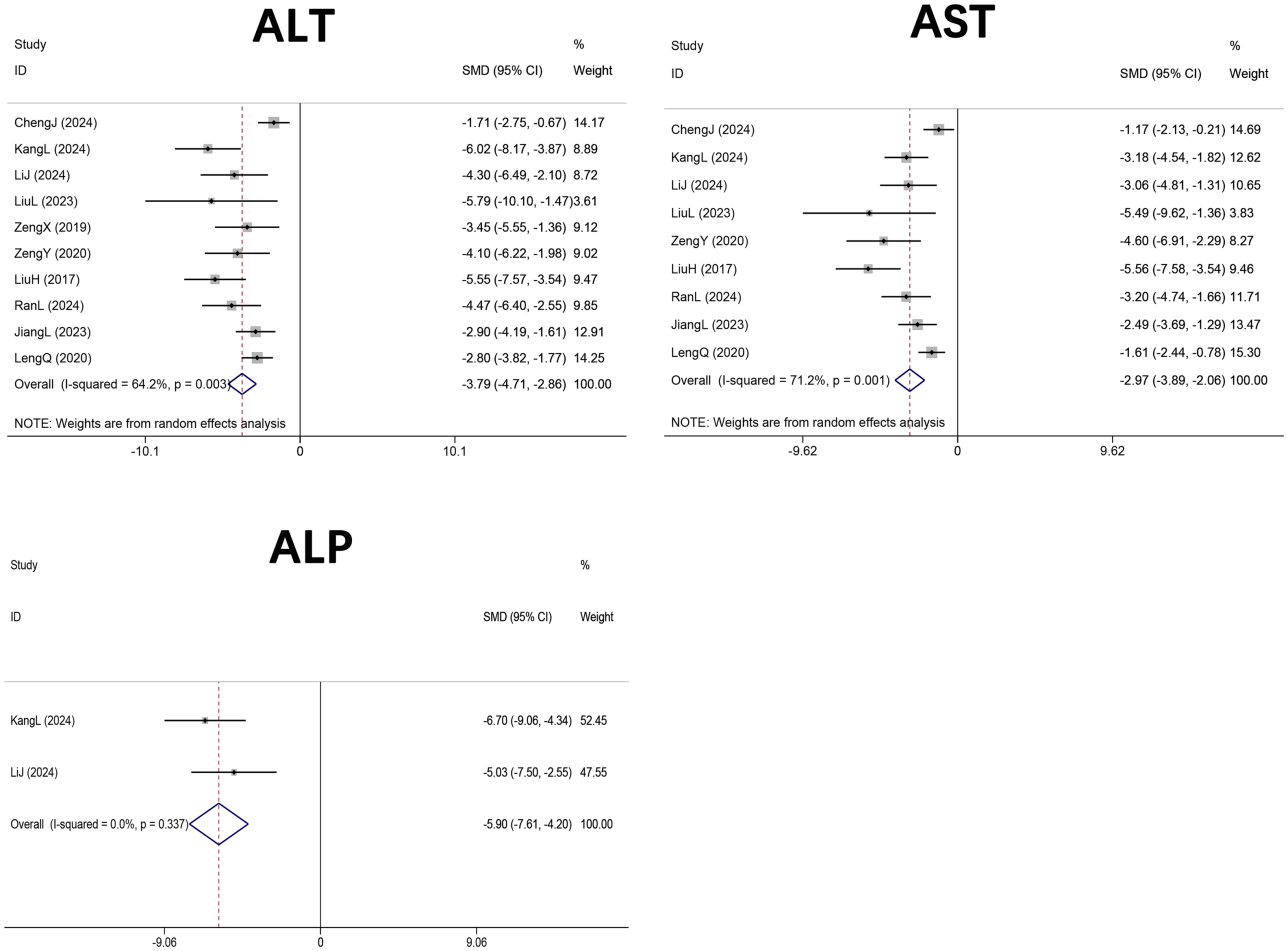
Forest plot of the effect of DHM versus control on liver enzymes in MASLD animal models. Effect sizes are shown as SMDs with 95% CIs based on random-effects meta-analysis. DHM, dihydromyricetin; MASLD, metabolic dysfunction-associated steatotic liver disease; ALT, alanine aminotransferase; AST, aspartate aminotransferase; ALP, alkaline phosphatase; SMD, standardized mean difference; 95% CI, confidence interval.

Ten studies (29, 30, 33, 43, 44, 46–48, 50, 51) (n = 166 mice) reported serum ALT, nine studies (30, 33, 43, 44, 46–48, 50, 51) (n = 156 mice) assessed AST, and two studies (30, 43) (*n* = 32 mice) evaluated ALP levels in HFD-induced NAFLD mice treated with DHM. Meta-analyses demonstrated significant reductions in ALT (SMD = −3.79, 95% CI: −4.71, −2.86, *p* < 0.001; I^2^ = 64.2%, *p* = 0.003), AST(SMD = −2.97, 95% CI: −3.89 to −2.06, *p* < 0.001; I^2^ = 71.2%, *p* = 0.001), and ALP (SMD = −5.77, 95% CI: −7.41 to −4.14, p < 0.001; I^2^ = 7.3%, *p* = 0.299) in DHM-treated groups compared with controls (Figure 6).

#### 3.4.5 Oxidative stress markers

**Figure 7:**
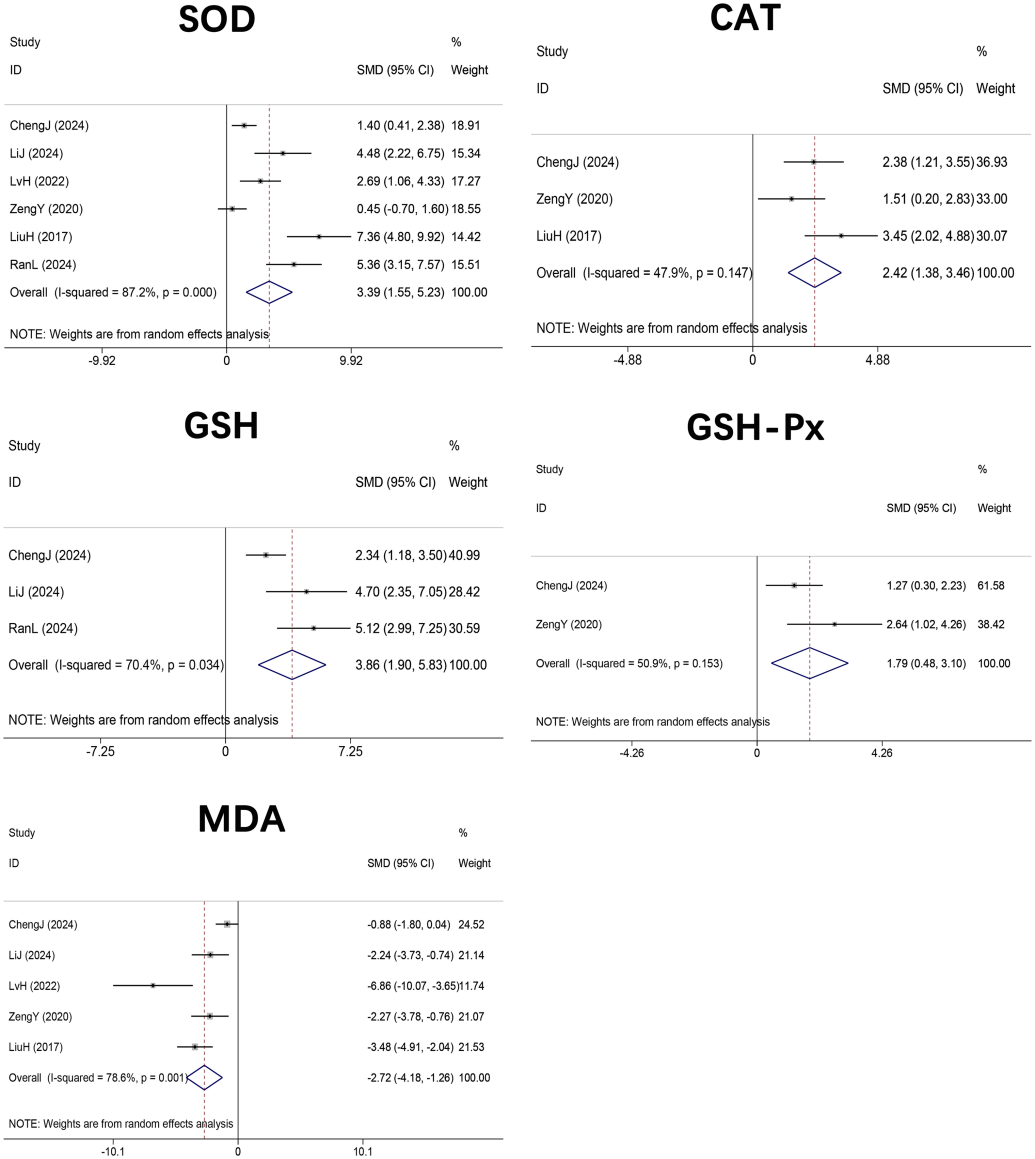
Forest plot of the effect of DHM versus control on oxidative stress markers in MASLD animal models. Effect sizes are shown as SMDs with 95% CI based on random-effects models. MASLD, metabolic dysfunction-associated steatotic liver disease; SMD, standardized mean difference; 95% CI, confidence interval; MDA, malondialdehyde; SOD, superoxide dismutase; GSH, glutathione; GSH-Px, glutathione peroxidase; CAT, catalase.

Six studies (33, 43, 45–48) (*n* = 96) evaluated SOD, three studies (33, 46, 47) (*n* = 52) assessed CAT, three studies (33, 43, 48) (*n* = 48) examined GSH, two studies (33, 46) (*n* = 32) measured GSH-Px, and five studies (33, 43, 45–47) (*n* = 80) determined MDA levels in hepatic tissue. Meta-analyses demonstrated significant increases in SOD (SMD = 3.39, 95% CI 1.55 to 5.23, *p* < 0.001; I^2^ = 87.2%, *p* < 0.001), CAT (SMD = 2.42, 95% CI 1.38 to 3.46, *p* < 0.001; I^2^ = 47.9%, *p* = 0.147), GSH (SMD = 3.86, 95% CI 1.90 to 5.83, *p* < 0.001; I^2^ = 70.4%, *p* = 0.034), and GSH-Px (SMD = 1.79, 95% CI 0.48 to 3.10, *p* = 0.007; I^2^ = 50.9%, *p* = 0.153), accompanied by a significant reduction in MDA (SMD = −2.72, 95% CI –4.18 to −1.26, *p* < 0.001; I^2^ = 78.6%, *p* = 0.001). These findings indicate that DHM markedly attenuates hepatic oxidative stress by enhancing antioxidant enzyme activities and reducing lipid peroxidation (Figure 7).

#### 3.4.6 Inflammatory cytokines

**Figure 8:**
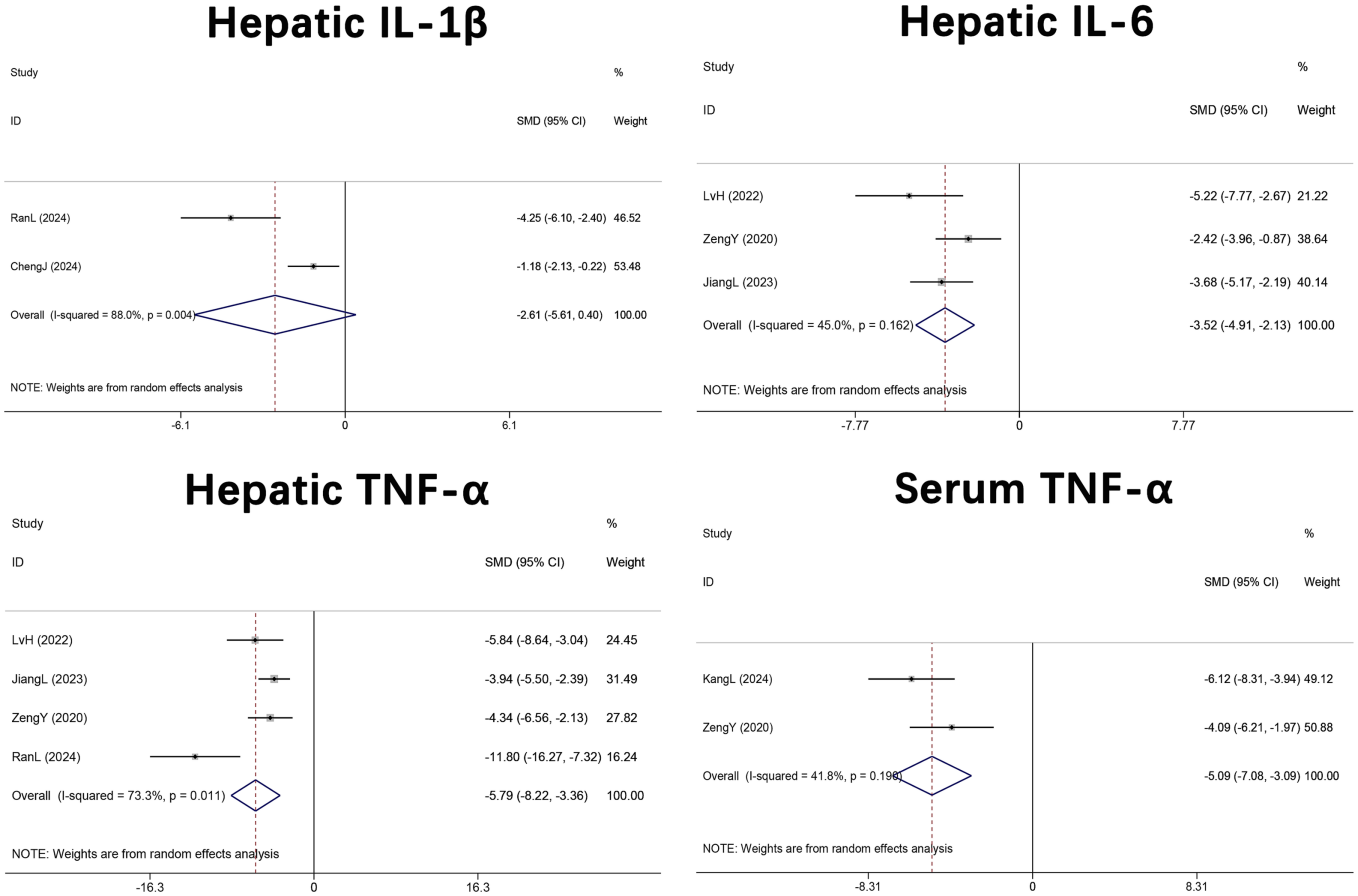
Forest plot of the effect of DHM versus control on inflammatory cytokines in MASLD animal models. Pooled SMDs with 95% CI were estimated using random-effects models. MASLD, metabolic dysfunction-associated steatotic liver disease; SMD, standardized mean difference; 95% CI, confidence interval; TNF, tumor necrosis factor; IL, interleukin.

Two studies (33, 48) (*n* = 36) reported hepatic IL-1β, three studies (45, 46, 50) (*n* = 44) assessed hepatic IL-6, four studies (45, 46, 48, 50) (*n* = 76) measured hepatic TNF-*α*, and two studies (30, 46) (*n* = 32) determined serum TNF-α levels. Meta-analyses revealed no significant change in IL-1β (SMD = −2.61, 95% CI –5.61 to 0.40, *p* = 0.089; I^2^ = 88.0%, *p* = 0.004), whereas IL-6 (SMD = −3.52, 95% CI –4.91 to −2.13, *p* < 0.001; I^2^ = 45.0%, *p* = 0.162), hepatic TNF-α (SMD = −5.79, 95% CI –8.22 to −3.36, *p* = 0.011; I^2^ = 88.9%, *p* < 0.001), and serum TNF-α (SMD = −5.09, 95% CI –7.08 to −3.09, *p* < 0.001; I^2^ = 73.3%, *p* = 0.011) were significantly reduced in DHM-treated mice. In sum, these findings suggest that DHM exerts potent anti-inflammatory effects both systemically and within hepatic tissue (Figure 8).

#### 3.4.7 Hepatic signaling proteins

Three studies (45, 48, 51) (*n* = 58) measured hepatic PPAR-α, two studies (45, 51) (*n* = 42) assessed CPT-1, and two studies (29, 46) (*n* = 22) examined pAMPK/AMPK ratio. Meta-analyses revealed no significant differences in PPAR-*α* (SMD = 2.91, 95% CI –1.20 to 7.02, *p* = 0.164; I^2^ = 94.7%, *p* < 0.001) or CPT-1 (SMD = 1.72, 95% CI –3.84 to 7.27, *p* = 0.545; I^2^ = 95.1%, *p* < 0.001), whereas the pAMPK/AMPK ratio was significantly increased in DHM-treated mice (SMD = 6.07, 95% CI 0.69 to 11.45, *p* = 0.033; I^2^ = 78.0%, *p* = 0.033). These molecular findings suggest that DHM may activate AMPK signaling, potentially contributing to its observed lipid-lowering and antioxidant effects (Supplementary Figure S12).

### 3.5 Subgroup analysis by intervention duration

To explore potential sources of heterogeneity, subgroup analyses were conducted according to intervention dose (> 200 mg/kg/day vs. ≤ 200 mg/kg/day) (Table 2) and treatment duration (> 8 weeks vs. ≤ 8 weeks) (Table 3). In dose-based analyses, high doses (> 200 mg/kg/day) were associated with greater improvements in hepatic TG, hepatic Tche, PPAR-*α*, MDA, TNF-α, body weight, liver index, serum lipid parameters, and insulin, whereas lower dose DHM (≤ 200 mg/kg/day) showed more pronounced effects on ALT, AST, FBG, and several oxidative stress markers, including SOD, CAT, and GSH. Heterogeneity decreased to varying degrees for most outcomes after dose stratification, with five outcomes (hepatic Tche, FBG, insulin, CAT, and GSH) exhibiting no observed heterogeneity (I^2^ = 0%).

**Table 2:** Subgroup analyses of dihydromyricetin on MASLD in mice by dosage.

Outcome	Dose	No. of studies	Heterogeneity (*p* value)	SMD	95%CI	p value for pooled effect
Hepatic TG	High	4	66.3% (0.031)	−3.76	−5.72, −1.80	<0.001
	Low	3	90.6% (<0.001)	−2.86	−5.47, −0.25	
Hepatic Tche	High	3	0.0% (0.871)	−2.63	−3.54, −1.72	<0.001
	Low	2	93.1% (<0.001)	−1.18	−4.77, 2.42	
Serum ALT	High	6	69.2% (0.006)	−3.61	−4.97, −2.26	0.003
	Low	4	57.9% (0.068)	−4.09	(−5.42,-2.76)	
Serum AST	High	5	69.6% (0.011)	−2.85	−4.14, −1.56	0.001
	Low	4	79.3% (0.002)	−3.20	−4.81, −1.59	
BW	High	8	84.5% (<0.001)	−2.81	−4.19, −1.43	<0.001
	Low	5	94.5% (<0.001)	−2.13	−4.71, 0.46	
liver index	High	4	59.4% (0.061)	−2.95	−4.32, −1.59	<0.001
	Low	2	96.5% (<0.001)	−2.26	−6.86, 2.34	
Serum TG	High	6	84.8% (<0.001)	−0.95	−2.34, 0.44	<0.001
	Low	5	89.5% (<0.001)	−0.36	−1.84, 1.12	
Serum Tche	High	7	57.0% (0.030)	−2.82	−3.75, −1.89	<0.001
	Low	5	83.5% (<0.001)	−0.89	−2.00, 0.22	
Serum LDL	High	7	74.9% (0.001)	−2.34	−3.47, −1.20	<0.001
	Low	4	78.8% (<0.001)	−0.80	−2.35, 0.75	
Serum HDL	High	7	72.5% (0.001)	1.30	0.34, 2.27	<0.001
	Low	3	95.0% (<0.001)	1.46	−1.53, 4.44	
FBG	High	3	65.8% (0.054)	−1.08	−2.17, −0.02	0.002
	Low	2	0.0% (0.374)	−3.05	−3.82, −2.29	
Insulin	High	2	0.0% (0.374)	−2.47	−3.50, −1.45	0.027
	Low	1	–	−0.53	−1.26, 0.20	
Hepatic PPAR-α	High	1	–	5.72	2.97, 8.47	<0.001
	Low	2	95.5% (<0.001)	1.64	−2.91, 6.19	
Hepatic SOD	High	3	59.4% (0.085)	1.39	0.28, 2.51	<0.001
	Low	3	28.5% (0.247)	5.64	4.04, 7.24	
Hepatic CAT	High	2	0.0% (0.335)	2.00	1.12, 2.87	0.147
	Low	1	–	3.45	2.02, 4.88	
Hepatic GSH	High	1	–	2.34	1.18, 3.50	0.034
	Low	2	0.0% (0.795)	4.93	3.35, 6.51	
Hepatic MDA	High	3	85.1% (0.001)	−2.84	−5.30, −0.38	0.001
	Low	2	27.5% (0.240)	−2.88	−4.09, −1.66	
Hepatic TNF-α	High	3	76.6% (0.014)	−6.85	−10.46, −3.23	<0.001
	Low	1	–	−3.94	−5.50, −2.39	

SMD, standardized mean difference; 95% CI, confidence interval; TG, triglycerides; Tche, total cholesterol; ALT, alanine aminotransferase; AST, aspartate aminotransferase; BW, body weight, LDL, low-density lipoprotein; HDL, high-density lipoprotein; FBG, fasting blood glucose; SOD, superoxide dismutase; CAT, catalase; GSH, glutathione; MDA, malondialdehyde; TNF, tumor necrosis factor.

**Table 3:** Subgroup analyses of dihydromyricetin on MASLD in mice by duration of intervention.

Outcome	Duration of intervention	No. of studies	Heterogeneity (*p* value)	SMD	95%CI	*p* value for pooled effect
Hepatic TG	Long	4	66.3% (0.031)	−3.76	−5.72, −1.80	<0.001
	Short	3	90.6 (<0.001)	−2.86	−5.47, −0.25	
Hepatic Tche	Long	3	0.0% (0.871)	−2.63	−3.54, −1.72	<0.001
	Short	2	93.1% (<0.001)	−1.18	−4.77, 2.42	
Serum ALT	Long	5	66.3% (0.011)	−3.53	−4.81, −2.25	0.003
	Short	4	64.1% (0.039)	−4.21	−5.67, −2.74	
Serum AST	Long	6	77.1% (0.001)	−3.37	−4.84, −1.91	0.001
	Short	3	64.1% (0.062)	−2.53	−3.71, −1.36	
BW	Long	8	89.4% (<0.001)	−2.09	−3.48, −0.69	<0.001
	Short	5	93.1% (<0.001)	−3.99	−7.05, −0.93	
Liver index	Long	1	–	−3.50	−4.94, −2.06	<0.001
	Short	4	89.2% (<0.001)	−2.67	−4.69, −0.65	
Serum TG	Long	8	82.7% (<0.001)	−1.20	−2.33, −0.07	<0.001
	Short	5	89.2% (<0.001)	0.13	−1.61, 1.87	
Serum Tche	Long	8	69.0% (0.002)	−2.04	−2.88, −1.20	0.002
	Short	4	91.9% (<0.001)	−1.91	−4.17, 0.36	
Serum LDL	Long	8	85.6% (<0.001)	−1.62	−2.83, −0.42	<0.001
	Short	3	67.2% (0.047)	−2.08	−3.43, −0.72	
Serum HDL	Long	3	75.4% (0.017)	1.21	−0.20, 2.61	<0.001
	Short	7	89.2% (<0.001)	1.50	0.01, 2.98	
FBG	Long	4	84.5% (<0.001)	−1.69	−3.10, −0.27	0.002
	Short	1	–	−2.75	−3.77, −1.73	
Hepatic PPAR-α	Long	1	–	5.72	2.97, 8.47	<0.001
	Short	2	95.5% (<0.001)	1.64	−2.91, 6.19	
Hepatic SOD	Long	4	88.1% (<0.001)	2.68	0.61, 4.74	<0.001
	Short	2	0.0% (<0.001)	4.93	3.35, 6.52	
Hepatic GSH	Long	1	–	2.34	1.18, 3.50	0.034
	Short	2	0.0% (0.795)	4.93	3.35, 6.51	
Hepatic MDA	Long	4	83.9% (<0.001)	−2.95	−4.86, −1.04	0.001
	Short	1	–	−2.24	−3.73, −0.74	
Hepatic TNF-α	Long	3	0.0% (0.510)	−4.38	−5.54, −3.22	<0.001
	Short	1	–	−1.80	−16.27, −7.32	

SMD, standardized mean difference; 95% CI, confidence interval; TG, triglycerides; Tche, total cholesterol; ALT, alanine aminotransferase; AST, aspartate aminotransferase; BW, body weight; LDL, low-density lipoprotein; HDL, high-density lipoprotein; FBG, fasting blood glucose; SOD, superoxide dismutase; GSH, glutathione; MDA, malondialdehyde; TNF, tumor necrosis factor.

When stratified by intervention duration, long-term treatment (> 8 weeks) generally produced more stable and consistent benefits compared with short-term interventions (≤ 8 weeks). For example, heterogeneity for hepatic Tche decreased from I^2^ = 82.4% overall to 0% within the long-term subgroup. Additionally, significant improvements in hepatic TG, TNF-α, serum TG, serum Tche, and HDL were observed only in the long-term group. In contrast, several outcomes—such as liver index and FBG— remained heterogeneous despite duration-based stratification (Table 3).

## 4 Discussion

### 4.1 Principal findings and possible mechanisms

This preclinical meta-analysis synthesized data from 14 murine studies and provides the first consolidated evidence that DHM exerts multidimensional hepatoprotective effects in diet-induced NAFLD models. Overall, DHM significantly attenuated hepatic lipid accumulation, lowered circulating atherogenic lipids, improved liver injury biomarkers (ALT, AST, ALP), reduced body weight and liver index, ameliorated glucose dysregulation, and enhanced antioxidant and anti-inflammatory responses. Taken together, these findings suggest that DHM does not act through a single pathway but rather through a coordinated modulation of metabolic, redox, and inflammatory networks, supporting its candidacy as a multi-target natural compound for MASLD. The following sections discuss these findings along the MASLD progression cascade, from metabolic overload and steatosis to hepatocellular injury and upstream signaling regulation.

### 4.2 Lipid metabolism and hepatic steatosis

A major observation of this study is that DHM improved both hepatic and systemic lipid homeostasis. On the hepatic side, pooled analyses supported large standardized effects on liver triglyceride and total cholesterol, indicating a genuine anti-steatotic action at the organ level. On the systemic level, DHM consistently lowered serum total cholesterol and LDL while increasing HDL; however, serum triglycerides did not change significantly—a pattern that has also been reported for other polyphenolic and flavonoid interventions in metabolic liver disease. This lipid-modulating profile is biologically plausible. DHM has been shown to activate AMPK, which in turn suppresses *de novo* lipogenesis by inhibiting ACC/FAS and, in parallel, may downregulate hepatic cholesterol synthesis through HMGCR inhibition and promote LDL clearance via LDLR/PCSK9-related pathways (57–66). The lack of significantly pooled increases in PPAR-*α* or CPT-1 expression in our analysis likely reflects the small number of available studies and methodological variability rather than a true absence of DHM action on fatty acid oxidation. In other words, the phenotypic outcomes—lower hepatic TG, lower serum Tche and LDL, and higher HDL—are more consistent than the molecular readouts—a phenomenon commonly observed in preclinical NAFLD meta-analyses due to variable assay sensitivity and reporting practices.

### 4.3 Anthropometric parameters

In addition to the biochemical markers, the observed reductions in body weight and liver index after DHM treatment are significant. These anthropometric parameters are widely recognized as early indicators of metabolic dysfunction and liver pathologies in high-fat diet-induced NAFLD models (67–69). Body weight increases and liver enlargement (reflected by liver index) are commonly associated with excessive lipid accumulation in the liver, a key feature of the early to middle stages of NAFLD. The reduction in body weight and liver index following DHM administration suggests that DHM may intervene early in the disease process by reducing metabolic overload and preventing further liver enlargement. Mechanistically, these changes are likely linked to the modulation of lipid metabolism pathways, particularly through the activation of AMPK. AMPK activation inhibits lipogenesis by suppressing ACC/FAS activity and enhances fatty acid oxidation, leading to a reduction in hepatic triglyceride accumulation (70, 71). Additionally, AMPK activation may improve mitochondrial function, which further contributes to a reduction in lipid deposition and liver enlargement (72–74).

### 4.4 Glucose metabolism

DHM also improved weight-related and glycometabolic parameters, which are pathophysiologically relevant to MASLD. In high-fat diet–induced mouse models, reductions in body weight and liver index are typically parallel to decreases in hepatic fat deposition and low-grade inflammation (75, 76). Consistent with this, our pooled effects on fasting glucose and insulin suggest that DHM may alleviate insulin resistance at both hepatic and peripheral levels. Mechanistically, these findings align with prior evidence showing DHM-induced activation of AMPK and insulin receptor substrate-1/protein kinase B (IRS-1/AKT) signaling, suppression of gluconeogenic enzymes such as PEPCK and G6Pase, and, in some reports, improvements in mitochondrial function and the promotion of adipose tissue browning (57, 77–85). Taken together, these converging actions position DHM among plant-derived compounds that target the “metabolic core” of MASLD rather than acting solely through antioxidant pathways.

### 4.5 Liver injury

DHM treatment significantly reduced liver injury biomarkers (ALT, AST, ALP), suggesting its potential hepatoprotective effects. These findings align with the known role of oxidative stress and inflammation in the progression of NAFLD (86–89). DHM’s ability to reduce liver injury is likely mediated through its antioxidant and anti-inflammatory properties. Mechanistically, DHM has been shown to enhance antioxidant defenses by increasing SOD, CAT, GSH, and GSH-Px, and by reducing MDA levels, which mitigates lipid peroxidation and oxidative damage to hepatocytes (90–93). This action likely prevents hepatocellular injury and liver dysfunction. In parallel, DHM downregulated key inflammatory mediators, including TNF-*α* and IL-6, both of which play a central role in liver damage and fibrosis in NAFLD (94–96). By suppressing these pro-inflammatory cytokines, DHM may help reduce liver inflammation, a critical component in the progression of the disease (97, 98). These findings suggest that DHM’s hepatoprotective effects are mediated by a combination of oxidative stress attenuation and inflammatory cytokine suppression, which are key to alleviating hepatocellular injury and potentially preventing further disease progression in NAFLD. Future studies should explore the role of other signaling pathways, such as Nrf2, to fully elucidate the molecular mechanisms underlying these protective effects (99–101).

### 4.6 Oxidative stress and inflammatory cytokines

Another important finding is the concurrent reinforcement of antioxidant defenses and suppression of inflammatory mediators. DHM markedly increased SOD, CAT, GSH, and GSH-Px and reduced MDA, indicating mitigation of lipid peroxidation and restoration of redox balance. This antioxidant pattern is consistent with activation of the canonical Keap1–Nrf2–heme oxygenase-1 (HO-1) axis, a mechanism frequently reported for DHM in extrahepatic models (102–110). In parallel, DHM significantly downregulated hepatic and circulating TNF-*α* and hepatic IL-6, both of which are key drivers of hepatocellular injury, insulin resistance, and fibrogenic signaling in MASLD. The absence of a significant pooled effect on IL-1β likely reflects the small number of available studies and wide confidence intervals, rather than a true lack of biological response. Overall, these data support a mechanistic model in which DHM interrupts the oxidative-stress–inflammation vicious cycle: activation of Nrf2 and improved mitochondrial function reduce ROS generation, which subsequently dampens NF-κB/NLRP3 activity and cytokine release (103, 104, 111–113). This coordinated antioxidant and anti-inflammatory signature may represent a comparative advantage of DHM over other flavonoids that target predominantly either lipid metabolism or oxidative stress alone (114–116).

### 4.7 Hepatic signaling proteins

The activation of hepatic signaling molecules plays a key role in DHM’s hepatoprotective effects. Our meta-analysis revealed that DHM significantly increased the pAMPK/AMPK ratio (SMD = 6.07, 95% CI 0.69–11.45), supporting activation of AMPK signaling, which is crucial for regulating lipid metabolism, inflammation, and oxidative stress (117–119). AMPK activation suppresses *de novo* lipogenesis by inhibiting ACC and FAS and promotes fatty acid oxidation, which contributes to the reduction of hepatic triglycerides and serum cholesterol levels (120, 121). This finding aligns with previous studies indicating that AMPK activation improves mitochondrial function and increases oxidative phosphorylation, thereby contributing to enhanced antioxidant defenses (122, 123). However, no significant changes were observed in PPAR-*α* and CPT-1 expression, which could reflect the relatively small number of studies and methodological variability in assessing these markers. PPAR-α is a critical regulator of fatty acid oxidation, and its activation is commonly associated with improved lipid profiles (124–126). The lack of significant changes in PPAR-α expression may be due to variations in experimental conditions or the insufficient number of studies available for this marker. Similarly, the lack of significant effects on CPT-1, a key enzyme in mitochondrial fatty acid oxidation, may indicate that DHM’s effects on fatty acid metabolism could be mediated through other pathways, such as AMPK signaling (127–129).

In addition to AMPK-centered signaling, mTORC1 represents a complementary nutrient-sensing hub that is functionally linked to several outcome domains observed in our analysis. Notably, AMPK is a well-established upstream negative regulator of mTORC1, and the increased pAMPK/AMPK ratio (i.e., AMPK activation) observed across included studies is conceptually consistent with restrained mTORC1 activity (e.g., via TSC2 and/or Raptor phosphorylation) (130–132). However, because most included studies assessed pathway markers without pharmacological inhibition or genetic perturbation, this AMPK–mTORC1 linkage should be interpreted as mechanistic plausibility rather than confirmed causality. Such AMPK–mTOR antagonism is pathophysiologically relevant to MASLD, as mTORC1 overactivation promotes anabolic lipid programs and suppresses autophagy, whereas its inhibition favors metabolic stress adaptation (133, 134). This AMPK–mTOR nutrient-sensing axis has been increasingly emphasized in recent MASLD-related mechanistic studies, where mTORC1 signaling is described as a central regulator of lipid synthesis, autophagy suppression, and metabolic transcriptional programs (133, 134). In addition, persistent mTORC1 activation has been linked to hyperinsulinemia-driven steatotic progression through PDX1-dependent endocrine mechanisms (135), while coordinated AMPK/SIRT1–mTOR balance is considered critical for mitochondrial quality control and metabolic stress resistance (130). This framework may also be indirectly consistent with our pooled findings showing improved oxidative stress markers (increased SOD, CAT, and GSH with reduced MDA), since mTORC1 suppression is associated with restored autophagic flux and mitochondrial quality control (130, 133). In addition, mTOR signaling has been implicated in inflammatory metabolic programming (133, 136, 137), providing a plausible upstream context for the observed reductions in TNF-*α* and IL-6, although causal linkage cannot be established from the current data.

These molecular findings suggest that DHM’s beneficial effects in NAFLD may be at least in part mediated by AMPK signaling, contributing to its lipid-lowering and antioxidant actions. While further studies are needed to fully elucidate the role of PPAR-α and CPT-1, the significant increase in pAMPK/AMPK ratio supports the idea that DHM appears to target key metabolic pathways involved in lipid metabolism and oxidative stress.

Importantly, the mechanistic signals identified in the included studies appear to follow a hierarchical pattern rather than representing isolated pathways. AMPK activation is likely positioned upstream, given its central role in regulating lipid metabolism, mitochondrial function, and oxidative stress responses (117, 118, 138). Downstream changes in antioxidant defense systems (e.g., SOD, CAT, GSH-related enzymes) and inflammatory mediators (TNF-α, IL-6) may therefore represent secondary effects of metabolic reprogramming rather than independent primary targets. However, because most included studies measured pathway markers without functional blockade experiments, these relationships should be interpreted as mechanistic associations rather than confirmed causal chains.

### 4.8 Heterogeneity

Notably, several pooled effects expressed as SMDs were large in magnitude. In preclinical animal studies, SMDs can be inflated when within-study variability is small (e.g., homogeneous strains, standardized housing and assays) or when methodological and measurement differences across laboratories affect the dispersion of outcomes (139). Therefore, the absolute magnitude of SMDs should be interpreted cautiously; we primarily emphasize the consistency in the direction of effects across studies and the concordant improvements observed across multiple biological domains, while acknowledging substantial between-study heterogeneity.

Nevertheless, the present meta-analysis revealed moderate-to-high heterogeneity across several outcomes. Our subgroup analyses indicated that both dose (>200 mg/kg/day vs. ≤ 200 mg/kg/day) and treatment duration (>8 weeks vs. ≤8 weeks) contributed meaningfully to this variability. Higher doses tended to yield stronger improvements in hepatic steatosis, circulating lipids, body weight, and inflammatory markers, whereas lower doses sometimes elicited more pronounced improvements in liver enzymes and certain antioxidant indices. Similarly, longer intervention periods yielded more consistent and homogeneous benefits; for several outcomes (e.g., hepatic Tche, FBG, insulin, CAT, and GSH), heterogeneity dropped to 0% once stratified by duration. This pattern suggests that DHM may require sufficient exposure time to fully engage metabolic and inflammatory pathways, while some redox-related responses may occur earlier during treatment. Residual heterogeneity after stratification is likely attributable to inherent variations in study design, including differences in mouse strain (C57BL/6 J vs. LDLR^−^/^−^ vs. ApoE^−^/^−^), diet composition, DHM formulation and purity, housing conditions, and the generally incomplete reporting of randomization and blinding. Such methodological inconsistencies are well recognized in preclinical MASLD research and have been repeatedly emphasized in ARRIVE and SYRCLE guidance (140, 141).

In addition, differences in administration route may also have contributed to heterogeneity. Most studies administered DHM by oral gavage, whereas one study delivered DHM through drinking water with an estimated dose conversion. Variations in administration route can influence absorption kinetics, effective exposure, and dose precision, thereby potentially affecting outcome variability. Although sensitivity analyses suggested that the estimated-dose study did not materially alter pooled results, route-related pharmacokinetic differences remain a plausible contributor to between-study heterogeneity. Taken together, these factors indicate that heterogeneity in the present meta-analysis is largely explainable by biological and design-related variability rather than inconsistent treatment direction. In addition, because data from the highest therapeutically tested dose per study were selected according to a prespecified rule, the pooled effect sizes may reflect upper-bound efficacy estimates rather than average-dose effects, and should therefore be interpreted conservatively. Although subgroup analyses were performed, substantial residual heterogeneity persisted for some outcomes. Therefore, these pooled estimates—particularly for outcomes with substantial residual heterogeneity—should be interpreted with caution, as unresolved between-study variability may reduce the robustness and precision of the estimates.

### 4.9 Limitations and future directions

Despite these encouraging findings, several limitations should be acknowledged. First, although 14 studies were included, some endpoints (insulin, IL-6, CAT, signaling proteins) were reported by only two or three experiments, which reduces precision and makes these outcomes more susceptible to small-study effects.

Second, while all studies included in this meta-analysis were conducted in China using male high-fat diet mouse models, this homogeneity in animal models enhances comparability and reduces experimental variability. However, it may limit the generalizability of the results, particularly when extrapolating findings to female animals, different MASLD models (e.g., Western-diet, MCD, or metabolic/hypertensive comorbid models), or diverse racial/regional populations. Previous research has shown that metabolic responses, including lipid accumulation and inflammation, may vary not only due to sex and genetic background, but also based on the disease model used. For instance, different models of diet-induced steatosis may result in distinct disease progression patterns, affecting how treatments like DHM are evaluated (56, 142–144). Given these considerations, future studies should aim to include female animal models, models of advanced disease stages, and models from diverse geographical backgrounds to better reflect the full spectrum of human disease progression and treatment responses. This restricted biological diversity may also contribute to variability in effect size estimates and treatment responsiveness, since both sex differences and model severity are known modifiers of metabolic and inflammatory outcomes in MASLD.

Third, substantial between-study heterogeneity was observed for several outcomes, which is a common feature of preclinical meta-analyses and may limit the precision of some pooled estimates. Despite this, effect directions were generally consistent across outcomes, and leave-one-out analyses indicated that no single study unduly drove the overall findings, supporting the overall robustness of the findings.

DHM is a food-derived flavonoid predominantly found in vine tea (*Ampelopsis grossedentata*), with reported high abundance in dried plant material, which supports its nutraceutical appeal (145). However, DHM exhibits limited oral bioavailability due to solubility/instability and rapid metabolism, and formulation or delivery strategies may be required to achieve sustained systemic exposure (146). Notably, human studies in metabolic liver disease have used capsule-based supplementation regimens (e.g., 150 mg capsules administered twice daily for 3 months), and early-phase safety/pharmacokinetic dose-escalation studies are ongoing, suggesting practical feasibility of supplement-level dosing while highlighting the need for further optimization of formulation and long-term safety evaluation (82).

Another important limitation relates to mechanistic inference. Most included studies evaluated pathway activity using expression or phosphorylation markers without employing pathway inhibition, pharmacological blockade, or genetic manipulation models (e.g., knockout or knockdown designs). Therefore, although AMPK-, Nrf2-, and inflammation-related pathways were consistently associated with DHM treatment effects, direct causal relationships cannot be firmly established. Future mechanistic studies using pathway-specific inhibition or genetic models are needed to validate mechanistic hierarchy and causality.

In view of these findings, DHM may be regarded as a promising, mechanistically plausible, and biologically active candidate compound for MASLD; however, it remains at the preclinical stage of the translational pathway. Future research should prioritize (i) standardized animal studies employing predefined DHM doses, at least two treatment durations (≤8 weeks and ≥12 weeks), and harmonized MASLD outcome panels; (ii) mechanistic investigations that directly link AMPK/Nrf2/SIRT1 activation to histological and metabolic improvement; and (iii) early-phase clinical trials or rigorously controlled nutraceutical studies in patients with MASLD to determine whether the biochemical and inflammatory benefits observed in mice translate to humans. Well-designed translational studies and clinical trials are ultimately required to establish whether the preclinical advantages of DHM can be reproduced in MASLD patients.

## 5 Conclusion

This systematic review and preclinical meta-analysis suggests that DHM confers promising benefits across steatosis-related, metabolic, oxidative stress, and inflammatory endpoints in murine models of NAFLD. These findings support DHM as a multifaceted hepatoprotective candidate with mechanistic plausibility and potential translational value. While additional work is needed to establish its translational relevance, the current evidence base strengthens the rationale for continued investigation of DHM in metabolic liver disease. However, the high variability observed in some outcomes suggests that further research with more standardized protocols is necessary to confirm these findings. The translational relevance to humans remains uncertain due to species differences and methodological variability in the current preclinical evidence. In conclusion, while the evidence from this meta-analysis supports the potential benefits of DHM in the treatment of NAFLD/MASLD, future studies should address the limitations regarding sex bias and geographical variability (142, 143). Expanding research to include female animal models and more diverse populations will be essential for improving the generalizability and clinical applicability of DHM treatment (56, 144).

## Data Availability

The original contributions presented in the study are included in the article/Supplementary material, further inquiries can be directed to the corresponding authors.

## Glossary

95% CI: 95% confidence interval
ACC: acetyl-CoA carboxylase
ALP: alkaline phosphatase
ALT: alanine aminotransferase
AMPK: AMP-activated protein kinase
AST: aspartate aminotransferase
BW: body weight
CAT: catalase
CPT-1: carnitine palmitoyltransferase-1
DHM: dihydromyricetin
EMA: European Medicines Agency
FAS: fatty acid synthase
FBG: fasting blood glucose
FDA: Food and Drug Administration
G6Pase: glucose-6-phosphatase
GSH: glutathione
GSH-Px: glutathione peroxidase
HDL: high-density lipoprotein cholesterol
HFD: high-fat diet
HMGCR: 3-hydroxy-3-methylglutaryl-CoA reductase
IL: interleukin
IRS-1/AKT: insulin receptor substrate-1/protein kinase B
Keap-1: Kelch-like ECH-associated protein 1
kg: kilogram
LDL: low-density lipoprotein cholesterol
MASLD: metabolic dysfunction-associated steatotic liver disease
MDA: malondialdehyde
mg: milligram
NA: not available
NAFLD: non-alcoholic fatty liver disease
NLRP3: NOD-like receptor family pyrin domain containing 3
Nrf2: nuclear factor erythroid 2-related factor 2
pAMPK: phosphorylated AMP-activated protein kinase
PEPCK: phosphoenolpyruvate carboxykinase
PPAR-*α*: peroxisome proliferator-activated receptor-α
PRISMA: Preferred Reporting Items for Systematic Reviews and Meta-Analyses
PROSPERO: International Prospective Register of Systematic Reviews
RoB: risk of bias
SD: standard deviation
SE: standard error
SMD: standardized mean difference
SOD: superoxide dismutase
SYRCLE: Systematic Review Centre for Laboratory Animal Experimentation
Tche: total cholesterol
TG: triglyceride
TNF-α: tumor necrosis factor-α
WMD: weighted mean difference

## References

[1] Fan X Song Y Zhao J. Evolving liver disease insights from NAFLD to MASLD. Trends Endocrinol Metab. (2024) 35:683–6. doi: 10.1016/j.tem.2024.02.012, 38429162

[2] Arshad T Golabi P Henry L Younossi ZM. Epidemiology of non-alcoholic fatty liver disease in North America. Curr Pharm Des. (2020) 26:993–7. doi: 10.2174/1381612826666200303114934, 32124690

[3] Majumdar A Verbeek J Tsochatzis EA. Non-alcoholic fatty liver disease: current therapeutic options. Curr Opin Pharmacol. (2021) 61:98–105. doi: 10.1016/j.coph.2021.09.007 34688168

[4] Arefhosseini S Ebrahimi-Mameghani M Najafipour F Tutunchi H. Non-alcoholic fatty liver disease across endocrinopathies: interaction with sex hormones. Front Endocrinol. (2022) 13:1032361. doi: 10.3389/fendo.2022.1032361, 36419770 PMC9676462

[5] Bellentani S Scaglioni F Marino M Bedogni G. Epidemiology of non-alcoholic fatty liver disease. Dig Dis. (2010) 28:155–61. doi: 10.1159/000282080, 20460905

[6] Brůha R. Non-alcoholic fatty liver disease. Vnitr Lek. (2019) 65:571–5. 31635468

[7] Han SK Baik SK Kim MY. Non-alcoholic fatty liver disease: definition and subtypes. Clin Mol Hepatol. (2023) 29:S5–s16. doi: 10.3350/cmh.2022.0424, 36577427 PMC10029964

[8] Ahmed MH Noor SK Bushara SO Husain NE Elmadhoun WM Ginawi IA . Non-alcoholic fatty liver disease in Africa and Middle East: An attempt to predict the present and future implications on the healthcare system. Gastroenterology Res. (2017) 10:271–9. doi: 10.14740/gr913w, 29118867 PMC5667692

[9] Akambase JA Prieto JE Mattos AZ Mattos AA Carrera E Díaz-Ferrer J . Epidemiology and risk factors for histopathologic characteristics of non-alcoholic fatty liver disease in South America. Aliment Pharmacol Ther. (2023) 58:526–36. doi: 10.1111/apt.17615, 37349900

[10] Golabi P Owrangi S Younossi ZM. Global perspective on nonalcoholic fatty liver disease and nonalcoholic steatohepatitis - prevalence, clinical impact, economic implications and management strategies. Aliment Pharmacol Ther. (2024) 59 Suppl 1:S1–s9. doi: 10.1111/apt.17833, 38813821

[11] Younossi ZM Koenig AB Abdelatif D Fazel Y Henry L Wymer M. Global epidemiology of nonalcoholic fatty liver disease-meta-analytic assessment of prevalence, incidence, and outcomes. Hepatology. (2016) 64:73–84. doi: 10.1002/hep.28431 26707365

[12] Lu H Zeng L Liang B Shu X Xie D. High prevalence of coronary heart disease in type 2 diabetic patients with non-alcoholic fatty liver disease. Arch Med Res. (2009) 40:571–5. doi: 10.1016/j.arcmed.2009.07.009, 20082871

[13] Bhatt SP Misra A Pandey RM. rs7903146 (C/T) polymorphism of transcription factor 7 like 2 (TCF7L-2) gene is independently associated with non-alcoholic fatty liver disease in Asian Indians. Diabetes Metab Syndr. (2020) 14:175–80. doi: 10.1016/j.dsx.2020.02.011 32142998

[14] Lattuada G Ragogna F Perseghin G. Why does NAFLD predict type 2 diabetes? Curr Diab Rep. (2011) 11:167–72. doi: 10.1007/s11892-011-0190-2, 21431854

[15] Jin D Jin S Zhou T Cui Z Guo B Li G . Regional variation in NAFLD prevalence and risk factors among people living with HIV in Europe: a meta-analysis. Front Public Health. (2023) 11:1295165. doi: 10.3389/fpubh.2023.1295165, 38259755 PMC10802187

[16] Jin D Cui Z Jin S Zhou T Guo B Gao P . Comparison of efficacy of anti-diabetics on non-diabetic NAFLD: a network meta-analysis. Front Pharmacol. (2022) 13:1096064. doi: 10.3389/fphar.2022.1096064, 36699084 PMC9868463

[17] Torres-Peña JD Martín-Piedra L Fuentes-Jiménez F. Statins in non-alcoholic steatohepatitis. Front Cardiovasc Med. (2021) 8:777131. doi: 10.3389/fcvm.2021.777131, 34901236 PMC8652077

[18] Pacana T Sanyal AJ. Recent advances in understanding/management of non-alcoholic steatohepatitis. F1000Prime Rep. (2015) 7:28. doi: 10.12703/p7-28, 25926979 PMC4371374

[19] Page JM Harrison SA. NASH and HCC. Clin Liver Dis. (2009) 13:631–47. doi: 10.1016/j.cld.2009.07.007 19818310

[20] Drygalski K. Pharmacological treatment of MASLD: contemporary treatment and future perspectives. Int J Mol Sci. (2025) 26:518. doi: 10.3390/ijms26136518, 40650294 PMC12249978

[21] Barbhuiya PA Talukdar S Mondal NS Kumari P Ahmed M Pathak MP. Marine species, metabolites and macromolecules as potential therapeutics against obesity and metabolic dysfunction associated Steatotic liver disease (MASLD): a comprehensive review. Curr Top Med Chem. (2025) 25:43. doi: 10.2174/0115680266370869250630062043, 40676792

[22] Mellemkjær A Kjær MB Haldrup D Grønbæk H Thomsen KL. Management of cardiovascular risk in patients with metabolic dysfunction-associated steatotic liver disease. Eur J Intern Med. (2024) 122:28–34. doi: 10.1016/j.ejim.2023.11.012, 38008609

[23] Zacharia GS Gongati SR Kharel A Jacob A. Semaglutide in metabolic dysfunction-associated steatohepatitis: a narrative review. Cureus. (2025) 17:e95632. doi: 10.7759/cureus.95632, 41322896 PMC12660513

[24] Stine JG Rivas G Hummer B Duarte-Rojo A May CN Geyer N . Mobile health lifestyle intervention program leads to clinically significant loss of body weight in patients with NASH. Hepatol Commun. (2023) 7:52. doi: 10.1097/hc9.0000000000000052, 36930864 PMC10027041

[25] Jin D Jin S Zhou T Sheng G Gao P Li G. Effects of quercetin on metabolic dysfunction-associated Steatotic liver disease: a systematic review and Meta-analysis. Food Sci Nutr. (2025) 13:e71358. doi: 10.1002/fsn3.71358, 41404533 PMC12703814

[26] Ling H Zhu Z Yang J He J Yang S Wu D . Dihydromyricetin improves type 2 diabetes-induced cognitive impairment via suppressing oxidative stress and enhancing brain-derived neurotrophic factor-mediated neuroprotection in mice. Acta Biochim Biophys Sin. (2018) 50:298–306. doi: 10.1093/abbs/gmy003, 29425256

[27] Liu CM Yang W Ma JQ Yang HX Feng ZJ Sun JM . Dihydromyricetin inhibits Lead-induced cognitive impairments and inflammation by the adenosine 5'-monophosphate-activated protein kinase pathway in mice. J Agric Food Chem. (2018) 66:7975–82. doi: 10.1021/acs.jafc.8b02433, 29975840

[28] Kou X Liu X Chen X Li J Yang X Fan J . Ampelopsin attenuates brain aging of D-gal-induced rats through miR-34a-mediated SIRT1/mTOR signal pathway. Oncotarget. (2016) 7:74484–95. doi: 10.18632/oncotarget.12811, 27780933 PMC5342681

[29] Zeng X Yang J Hu O Huang J Ran L Chen M . Dihydromyricetin ameliorates nonalcoholic fatty liver disease by improving mitochondrial respiratory capacity and redox homeostasis through modulation of SIRT3 signaling. Antioxid Redox Signal. (2019) 30:163–83. doi: 10.1089/ars.2017.7172, 29310441

[30] Kang L Ma X Yu F Xu L Lang L. Dihydromyricetin alleviates non-alcoholic fatty liver disease by modulating gut microbiota and inflammatory signaling pathways. J Microbiol Biotechnol. (2024) 34:2637–47. doi: 10.4014/jmb.2406.06048, 39639497 PMC11729546

[31] Lyu Q Chen L Lin S Cao H Teng H. A designed self-microemulsion delivery system for dihydromyricetin and its dietary intervention effect on high-fat-diet fed mice. Food Chem. (2022) 390:132954. doi: 10.1016/j.foodchem.2022.132954, 35551031

[32] Leng Q Zhou J Li C Xu Y Liu L Zhu Y . Dihydromyricetin ameliorates diet-induced obesity and promotes browning of white adipose tissue by upregulating IRF4/PGC-1α. Nutr Metab. (2022) 19:72. doi: 10.1186/s12986-022-00672-6 PMC9188085 35690863

[33] Cheng J Huang SY Xiong RG Wu S-X Yang Z-J Zhou D-D . Vine tea kombucha ameliorates non-alcoholic fatty liver disease in high-fat diet fed mice via antioxidation, anti-inflammation and regulation of gut microbiota. Food Biosci. (2024) 62:105400. doi: 10.1016/j.fbio.2024.105400

[34] Gong H Xu H Li M Zhang D. Molecular mechanism and therapeutic significance of dihydromyricetin in nonalcoholic fatty liver disease. Eur J Pharmacol. (2022) 935:175325. doi: 10.1016/j.ejphar.2022.175325, 36265611

[35] Pereira MB Sydor BG Memare KG Verzignassi Silveira TG Alessi Aristides SM Dalmarco EM . In vivo efficacy of meglumine antimoniate-loaded nanoparticles for cutaneous leishmaniasis: a systematic review. Nanomedicine. (2021) 16:1505–18. doi: 10.2217/nnm-2021-0119, 34189952

[36] Osorio Parra MM Elangovan S Lee CT. Specialized pro-resolving lipid mediators in experimental periodontitis: a systematic review. Oral Dis. (2019) 25:1265–76. doi: 10.1111/odi.12979, 30230662

[37] Hao L Peng Q Li S Hu X Yan H. Novel insights from meta-analysis: the efficacy of ginsenosides in non-alcoholic fatty liver disease. Front Pharmacol. (2025) 16:1564852. doi: 10.3389/fphar.2025.1564852, 40495888 PMC12148916

[38] Zhang S Xue Y Zhang X Chen F Li Y Zhang W. Therapeutic effects and potential mechanisms of astragaloside IV on pulmonary fibrosis: a systematic review and meta-analysis of preclinical studies. Front Pharmacol. (2025) 16:1564290. doi: 10.3389/fphar.2025.1564290, 40822469 PMC12350305

[39] Ashcraft KA Peace RM Betof AS Dewhirst MW Jones LW. Efficacy and mechanisms of aerobic exercise on Cancer initiation, progression, and metastasis: a critical systematic review of in vivo preclinical data. Cancer Res. (2016) 76:4032–50. doi: 10.1158/0008-5472.Can-16-0887, 27381680 PMC5378389

[40] Ma B Xu JK Wu WJ Liu H-y Kou C-k Liu N . Survey of basic medical researchers on the awareness of animal experimental designs and reporting standards in China. PLoS One. (2017) 12:e0174530. doi: 10.1371/journal.pone.0174530, 28380050 PMC5381903

[41] Jin D Jin S Sheng G Cui Z Gao P Li G. Effects of curcumin on postmenopausal women's health: a systematic review and meta-analysis. Phytother Res. (2025) 39:2202–16. doi: 10.1002/ptr.8467, 40105038

[42] Li L Wu C Gan Y Qu X Lu Z. Insomnia and the risk of depression: a meta-analysis of prospective cohort studies. BMC Psychiatry. (2016) 16:375. doi: 10.1186/s12888-016-1075-3, 27816065 PMC5097837

[43] Li J Yin M Tian M Fang J Xu H. Stiff-soft hybrid biomimetic nano-emulsion for targeted liver delivery and treatment of early nonalcoholic fatty liver disease. Pharmaceutics. (2024) 16:303. doi: 10.3390/pharmaceutics16101303, 39458632 PMC11510375

[44] Liu L Shen Q Wang Y Li H Zhao J. Dihydromyricetin alleviates nonalcoholic fatty liver disease and its associated metabolic syndrome by inhibiting endoplasmic reticulum stress in LDLR−/−mice fed with a high-fat and high-fructose diet. J Clin Pharm Ther. (2023) 2023:934. doi: 10.1155/2023/5029934

[45] Lv H Xv T Peng J Luo G He JQ Yang S . Dihydromyricetin improves liver fat deposition in high-fat diet-induced obese mice. J Chin Pharm Sci. (2022) 31:824–39. doi: 10.5246/jcps.2022.11.070

[46] Zeng Y Hua YQ Wang W Zhang H Xu XL. Modulation of SIRT1-mediated signaling cascades in the liver contributes to the amelioration of nonalcoholic steatohepatitis in high fat fed middle-aged LDL receptor knockout mice by dihydromyricetin. Biochem Pharmacol. (2020) 175:113927. doi: 10.1016/j.bcp.2020.113927, 32217100

[47] Liu H. Protective effects and mechanism of dihydromyricetin on non-alcoholic fatty liver disease in ApoE−/− mice. Chin Tradit Patent Med. (2017) 39:2448–53. doi: 10.3969/j.issn.1001-1528.2017.12.002

[48] Ran L Yao Y Hou P Mi M Zhu J. Effect and mechanism of dihydromyricetin improving choline-deficient and L-amino acid-restricted high-fat diet-induced non-alcoholic steatohepatitis in mice. Occup Health. (2024) 40:2612–8.

[49] Ma X Chen K Ran L Zhu J Mi M. Relationship of mitochondrial fusion/fission genes with dihydromyricetin inhibiting liver fat accumulation in high-fat fed mice. J Third Mil Med Univ. (2018) 40:17–22. doi: 10.16016/j.1000-5404.201709101

[50] Jiang L. Dihydromyricetin Ameliorates Metabolic Associated Fatty Liver Disease by Regulating Gut Barrier Integrity through Gut Microbiota/ILC3 Pathway. [Master's thesis]. Chongqing: Army Medical University (2023).

[51] Leng Q Li C Zhou J Lu L Na L Sa G . Dihydromyricin ameliorates non-alcoholic fatty liver disease by inhibiting lipid synthesis. Int J Endocrinol Metab. (2020) 40:376–81. doi: 10.3760/cma.j.cn121383-20200205-02004

[52] Redelsperger IM Taldone T Riedel ER Lepherd ML Lipman NS Wolf FR. Stability of doxycycline in feed and water and minimal effective doses in tetracycline-inducible systems. J Am Assoc Lab Anim Sci. (2016) 55:467–74. 27423155 PMC4943619

[53] Marx JO Vudathala D Murphy L Rankin S Hankenson FC. Antibiotic administration in the drinking water of mice. J Am Assoc Lab Anim Sci. (2014) 53:301–6. 24827573 PMC4128569

[54] Bachmanov AA Reed DR Beauchamp GK Tordoff MG. Food intake, water intake, and drinking spout side preference of 28 mouse strains. Behav Genet. (2002) 32:435–43. doi: 10.1023/a:1020884312053, 12467341 PMC1397713

[55] OECD. Test No. 408: Repeated Dose 90-day Oral Toxicity Study in Rodents: OECD Guidelines for the Testing of Chemicals, Section 4. Paris: Organisation for Economic Co-operation and Development (OECD). (2018).

[56] Liu J Ding M Bai J Luo R Liu R Qu J . Decoding the role of immune T cells: a new territory for improvement of metabolic-associated fatty liver disease. iMeta. (2023) 2:e76. doi: 10.1002/imt2.76, 38868343 PMC10989916

[57] Yang Y Qiu W Xiao J Sun J Ren X Jiang L. Dihydromyricetin ameliorates hepatic steatosis and insulin resistance via AMPK/PGC-1α and PPARα-mediated autophagy pathway. J Transl Med. (2024) 22:309. doi: 10.1186/s12967-024-05060-7, 38532480 PMC10964712

[58] Liu Z Gan L Wu T Feng F Luo D Gu H . Adiponectin reduces ER stress-induced apoptosis through PPARα transcriptional regulation of ATF2 in mouse adipose. Cell Death Dis. (2016) 7:e2487. doi: 10.1038/cddis.2016.388, 27882945 PMC5260871

[59] Lee SK Lee JO Kim JH Kim N You GY Moon JW . Coenzyme Q10 increases the fatty acid oxidation through AMPK-mediated PPARα induction in 3T3-L1 preadipocytes. Cell Signal. (2012) 24:2329–36. doi: 10.1016/j.cellsig.2012.07.022, 22885103

[60] Fruchart JC Duriez P Staels B. Peroxisome proliferator-activated receptor-alpha activators regulate genes governing lipoprotein metabolism, vascular inflammation and atherosclerosis. Curr Opin Lipidol. (1999) 10:245–58. doi: 10.1097/00041433-199906000-00007, 10431661

[61] Nagasawa T Inada Y Nakano S Tamura T Takahashi T Maruyama K . Effects of bezafibrate, PPAR pan-agonist, and GW501516, PPARδ agonist, on development of steatohepatitis in mice fed a methionine- and choline-deficient diet. Eur J Pharmacol. (2006) 536:182–91. doi: 10.1016/j.ejphar.2006.02.028, 16574099

[62] Zhou W Han WF Landree LE Thupari JN Pinn ML Bililign T . Fatty acid synthase inhibition activates AMP-activated protein kinase in SKOV3 human ovarian cancer cells. Cancer Res. (2007) 67:2964–71. doi: 10.1158/0008-5472.Can-06-3439, 17409402

[63] Landree LE Hanlon AL Strong DW Rumbaugh G Miller IM Thupari JN . C75, a fatty acid synthase inhibitor, modulates AMP-activated protein kinase to alter neuronal energy metabolism. J Biol Chem. (2004) 279:3817–27. doi: 10.1074/jbc.M310991200, 14615481

[64] Kim JH Kang SI Shin HS Yoon SA Kang SW Ko HC . *Sasa quelpaertensis* and p-coumaric acid attenuate oleic acid-induced lipid accumulation in HepG2 cells. Biosci Biotechnol Biochem. (2013) 77:23832345:1595–8. doi: 10.1271/bbb.130167 23832345

[65] Loh K Tam S Murray-Segal L Huynh K Meikle PJ Scott JW . Inhibition of adenosine monophosphate-activated protein Kinase-3-Hydroxy-3-Methylglutaryl coenzyme a reductase signaling leads to hypercholesterolemia and promotes hepatic steatosis and insulin resistance. Hepatol Commun. (2019) 3:84–98. doi: 10.1002/hep4.1279, 30619997 PMC6312662

[66] Wang L-T Hu D-D Liu H-R Yin H-L Mu R Yang Y-H . Dihydromyricetin promotes LDL metabolism in HepG2 cells through the PCSK9/LDLR pathway. CyTA J Food. (2023) 21:554–60. doi: 10.1080/19476337.2023.2252044

[67] Le TNH Choi HJ Jun HS. Ethanol extract of *Liriope platyphylla* root attenuates non-alcoholic fatty liver disease in high-fat diet-induced obese mice via regulation of lipogenesis and lipid uptake. Nutrients. (2021) 13:338. doi: 10.3390/nu13103338, 34684339 PMC8538311

[68] Hu PA Chen CH Guo BC Kou YR Lee TS. Bromelain confers protection against the non-alcoholic fatty liver disease in male C57bl/6 mice. Nutrients. (2020) 12:458. doi: 10.3390/nu12051458, 32443556 PMC7285019

[69] Xu P Zhang XG Li YM Yu CH Xu L Xu GY. Research on the protection effect of pioglitazone for non-alcoholic fatty liver disease (NAFLD) in rats. J Zhejiang Univ Sci B. (2006) 7:627–33. doi: 10.1631/jzus.2006.B0627, 16845716 PMC1533756

[70] Chen K Chen X Xue H Zhang P Fang W Ling W. Coenzyme Q10 attenuates high-fat diet-induced non-alcoholic fatty liver disease through activation of the AMPK pathway. Food Funct. (2019) 10:814–23. doi: 10.1039/c8fo01236a, 30675881

[71] Zhang C Shi J Shi L. Natural products intervene in non-alcoholic fatty liver disease by regulating the AMPK signaling pathway: preclinical evidence and mechanism. Front Pharmacol. (2025) 16:1696506. doi: 10.3389/fphar.2025.1696506, 41383472 PMC12689597

[72] Ramanathan R Ali AH Ibdah JA. Mitochondrial dysfunction plays central role in nonalcoholic fatty liver disease. Int J Mol Sci. (2022) 23:7280. doi: 10.3390/ijms23137280, 35806284 PMC9267060

[73] Karkucinska-Wieckowska A Simoes IC Kalinowski P Simoes ICM Lebiedzinska-Arciszewska M Zieniewicz K . Mitochondria, oxidative stress and nonalcoholic fatty liver disease: a complex relationship. Eur J Clin Investig. (2022) 52:e13622. doi: 10.1111/eci.13622, 34050922

[74] Zheng Y Wang S Wu J Wang Y. Mitochondrial metabolic dysfunction and non-alcoholic fatty liver disease: new insights from pathogenic mechanisms to clinically targeted therapy. J Transl Med. (2023) 21:510. doi: 10.1186/s12967-023-04367-1, 37507803 PMC10375703

[75] Zheng Y Wu Y Tao L Chen X Jones TJ Wang K . Chinese propolis prevents obesity and metabolism syndromes induced by a high fat diet and accompanied by an altered gut microbiota structure in mice. Nutrients. (2020) 12:959. doi: 10.3390/nu12040959, 32235581 PMC7230861

[76] Xu S Deng Y Li C Hu Y Zhang Q Zhuang B . Metabolomics and molecular docking-directed anti-obesity study of the ethanol extract from Gynostemma pentaphyllum (Thunb.) Makino. J Ethnopharmacol. (2024) 334:118577. doi: 10.1016/j.jep.2024.118577, 39019414

[77] Tong H Zhang X Tan L Jin R Huang S Li X. Multitarget and promising role of dihydromyricetin in the treatment of metabolic diseases. Eur J Pharmacol. (2020) 870:172888. doi: 10.1016/j.ejphar.2019.172888, 31866404

[78] You J Zhao M Chen S Jiang L Gao S Yin H . Effect of chitooligosaccharides with a specific degree of polymerization on multiple targets in T2DM mice. Bioresour Bioprocess. (2022) 9:94. doi: 10.1186/s40643-022-00579-3, 38647883 PMC10992422

[79] Hou L Jiang F Huang B Zheng W Jiang Y Cai G . Dihydromyricetin ameliorates inflammation-induced insulin resistance via phospholipase C-CaMKK-AMPK signal pathway. Oxidative Med Cell Longev. (2021) 2021:8542809. doi: 10.1155/2021/8542809, 34650665 PMC8510796

[80] Shi L Zhang T Liang X Hu Q Huang J Zhou Y . Dihydromyricetin improves skeletal muscle insulin resistance by inducing autophagy via the AMPK signaling pathway. Mol Cell Endocrinol. (Jul 5 2015) 409:92–102. doi: 10.1016/j.mce.2015.03.009, 25797177

[81] Fayyaz S Qureshi MZ Alhewairini SS Avnioglu S Attar R Sabitaliyevich UY . Regulation of signaling pathways by Ampelopsin (Dihydromyricetin) in different cancers: exploring the highways and byways less travelled. Cell Mol Biol (Noisy-le-Grand). (2019) 65:15–20. doi: 10.14715/cmb/2019.65.7.4, 31880533

[82] Chen S Zhao X Wan J Ran L Qin Y Wang X . Dihydromyricetin improves glucose and lipid metabolism and exerts anti-inflammatory effects in nonalcoholic fatty liver disease: a randomized controlled trial. Pharmacol Res. (2015) 99:74–81. doi: 10.1016/j.phrs.2015.05.009, 26032587

[83] Yang C Du Y Wei L Tan Z Zhou T Wang L (2025);16:3613–3626. doi: 10.1039/d5fo01001b, 40232278. Preventive effects of turmeric against HFD/STZ-induced type 2 diabetes in mice by activating IRS1/PI3K/Akt signaling in association with gut microbiota metabolism. Food Funct. 40232278

[84] Li L Yao Y Zhao J Cao J Ma H. Dehydroepiandrosterone protects against hepatic glycolipid metabolic disorder and insulin resistance induced by high fat via activation of AMPK-PGC-1α-NRF-1 and IRS1-AKT-GLUT2 signaling pathways. Int J Obes. (2020) 44:1075–86. doi: 10.1038/s41366-019-0508-8, 31911660

[85] Zhang Y Chen J Zeng Y Huang D Xu Q. Involvement of AMPK activation in the inhibition of hepatic gluconeogenesis by *Ficus carica* leaf extract in diabetic mice and HepG2 cells. Biomed Pharmacother. (2019) 109:188–94. doi: 10.1016/j.biopha.2018.10.077, 30396076

[86] Lam P Cheung F Tan HY Wang N Yuen MF Feng Y. Hepatoprotective effects of Chinese medicinal herbs: a focus on anti-inflammatory and anti-oxidative activities. Int J Mol Sci. (2016) 17:465. doi: 10.3390/ijms17040465, 27043533 PMC4848921

[87] Zhang C-Y Liu S Yang M. Antioxidant and anti-inflammatory agents in chronic liver diseases: molecular mechanisms and therapy. World J Hepatol. (2023) 15:180–200. doi: 10.4254/wjh.v15.i2.180, 36926234 PMC10011909

[88] Farzanegi P Dana A Ebrahimpoor Z Asadi M Azarbayjani MA. Mechanisms of beneficial effects of exercise training on non-alcoholic fatty liver disease (NAFLD): roles of oxidative stress and inflammation. Eur J Sport Sci. (2019) 19:994–1003. doi: 10.1080/17461391.2019.1571114, 30732555

[89] Dias KA Oliveira LA Pereira SMS Abrantes LCS Vicente LCOS Gonçalves RV . Anti-inflammatory and antioxidant effects of anthocyanins in nonalcoholic fatty liver disease (NAFLD): a systematic review of in vivo studies. Crit Rev Food Sci Nutr. (2025) 65:7479–96. doi: 10.1080/10408398.2025.2472882, 40045715

[90] Liu S Shi P Jin Y Fan L Ni Q Mao J . Nicotinamide N-oxide alleviates sepsis-induced hepatic inflammation, oxidative stress, and mitochondrial damage depends on SIRT3/AKT signaling pathway. Toxicol Appl Pharmacol. (2026) 507:117709. doi: 10.1016/j.taap.2026.117709, 41506539

[91] Li W Chen H Xie Z Gao J Wang M Chen Z . Exploring the mechanism of dihydromyricetin in alleviating psoriasis based on metabolomics, network pharmacology and experimental validation. J Ethnopharmacol. (2026) 360:121167. doi: 10.1016/j.jep.2026.121167, 41539635

[92] Yu J Ao Y Chen H Liu C Deng T Wang D . Effects of dietary supplementation with dihydromyricetin on hindgut microbiota and metabolite profiles in dairy cows. Microorganisms. (2025) 14:20. doi: 10.3390/microorganisms14010020, 41597540 PMC12843980

[93] El-Hameed SSA Matouk AI Ibrahim ARN El-Daly M. Mechanistic insights into the anti-benign prostatic hyperplasia effect of dihydromyricetin via suppression of the 5-AR/TGF-β1/Smad2 axis. Naunyn Schmiedebergs Arch Pharmacol. (2026). doi: 10.1007/s00210-026-04998-3 [ahead of print]. PMC13152920 41629614

[94] Heitmann J Frings VG Geier A Goebeler M Kerstan A. Non-alcoholic fatty liver disease and psoriasis - is there a shared proinflammatory network? J Dtsch Dermatol Ges. (2021) 19:517–29. doi: 10.1111/ddg.14425, 33768700

[95] Khura J Khurana T Mehra S Singh P. Evaluation of pro-inflammatory markers IL-6 and TNF-a and their correlation with non-alcoholic fatty liver disease. J Adv Res Med. (2019) 6:1–6. doi: 10.24321/2349.7181.201906

[96] Vachliotis ID Polyzos SA. The role of tumor necrosis factor-alpha in the pathogenesis and treatment of nonalcoholic fatty liver disease. Curr Obes Rep. (2023) 12:191–206. doi: 10.1007/s13679-023-00519-y, 37407724 PMC10482776

[97] Petrescu M Vlaicu SI Ciumărnean L Milaciu MV Mărginean C Florea M . Chronic inflammation—a link between nonalcoholic fatty liver disease (NAFLD) and dysfunctional adipose tissue. Medicina. (2022) 58:641. doi: 10.3390/medicina58050641, 35630058 PMC9147364

[98] Sanyal AJ Williams SA Lavine JE Neuschwander-Tetri BA Alexander L Ostroff R . Defining the serum proteomic signature of hepatic steatosis, inflammation, ballooning and fibrosis in non-alcoholic fatty liver disease. J Hepatol. (2023) 78:693–703. doi: 10.1016/j.jhep.2022.11.029, 36528237 PMC10165617

[99] Zhou J Zheng Q Chen Z. The Nrf2 pathway in liver diseases. Front Cell Dev Biol. (2022) 10:826204. doi: 10.3389/fcell.2022.826204, 35223849 PMC8866876

[100] Echeverría F Bustamante A Sambra V Álvarez D Videla L Valenzuela R. Beneficial effects of dietary polyphenols in the prevention and treatment of NAFLD: cell-signaling pathways underlying health effects. Curr Med Chem. (2022) 29:299–328. doi: 10.2174/0929867328666210825111350, 34525916

[101] Yu W Zhang F Meng D Zhang X Feng Y Yin G . Mechanism of action and related natural regulators of nrf2 in nonalcoholic fatty liver disease. Curr Drug Deliv. (2024) 21:1300–19. doi: 10.2174/0115672018260113231023064614, 39034715

[102] Huang Y Chen K Ren Q Yi L Zhu J Zhang Q . Dihydromyricetin attenuates dexamethasone-induced muscle atrophy by improving mitochondrial function via the PGC-1α pathway. Cell Physiol Biochem. (2018) 49:758–79. doi: 10.1159/000493040, 30165349

[103] Hu Q Li C Zhang T Yi L Shan Y Ma X . Dihydromyricetin suppresses endothelial NLRP3 inflammasome activation and attenuates atherogenesis by promoting mitophagy. Lipids Health Dis. (2024) 23:279. doi: 10.1186/s12944-024-02263-1, 39227809 PMC11370113

[104] Jing N Li X. Dihydromyricetin attenuates inflammation through TLR4/NF-kappaB pathway. Open Med. (2019) 14:719–25. doi: 10.1515/med-2019-0083, 31572805 PMC6749725

[105] Wang X Li H Qu D. Dihydromyricetin protects sevoflurane-induced mitochondrial dysfunction in HT22 hippocampal cells. Clin Exp Pharmacol Physiol. (2024) 51:e13912. doi: 10.1111/1440-1681.13912, 39103220

[106] Yuan L Jiang X Ren Y Ma B Ji Z Wang S . Dihydromyricetin ameliorates lipopolysaccharide–induced hepatic injury in chickens by activating the Nrf2/Keap1 pathway and regulating mitochondrial dynamics. Poult Sci. (2025) 104:105034. doi: 10.1016/j.psj.2025.105034, 40132312 PMC11986534

[107] Hong H Guo D Xia T Zhang Y. Dihydromyricetin attenuates intervertebral disc degeneration by inhibiting NLRP3 inflammasome activation via the Keap1/Nrf2/HO-1 pathway. Eur J Pharmacol. (2025) 998:177501. doi: 10.1016/j.ejphar.2025.177501, 40058758

[108] Wei C Chen X Chen D Yu B Zheng P He J . Dihydromyricetin enhances intestinal antioxidant capacity of growing-finishing pigs by activating ERK/Nrf2/HO-1 signaling pathway. Antioxidants. (2022) 11:388. doi: 10.3390/antiox11040704, 35453388 PMC9028153

[109] Luo Y Lu S Dong X Xu L Sun G Sun X. Dihydromyricetin protects human umbilical vein endothelial cells from injury through ERK and Akt mediated Nrf2/HO-1 signaling pathway. Apoptosis. (2017) 22:1013–24. doi: 10.1007/s10495-017-1381-3, 28612103

[110] Zhang Q Wang J Zhang H Zeng T. Dihydromyricetin inhibits oxidative stress and apoptosis in oxygen and glucose deprivation/reoxygenation-induced HT22 cells by activating the Nrf2/HO-1 pathway. Mol Med Rep. (2021) 23:12036. doi: 10.3892/mmr.2021.12036, 33786616

[111] Hou X Tong Q Wang W Xiong W Shi C Fang J. Dihydromyricetin protects endothelial cells from hydrogen peroxide-induced oxidative stress damage by regulating mitochondrial pathways. Life Sci. (2015) 130:38–46. doi: 10.1016/j.lfs.2015.03.007, 25818185

[112] Zhang X Li X Fang J Hou X Fang H Guo F . (2R,3R)Dihydromyricetin inhibits osteoclastogenesis and bone loss through scavenging LPS-induced oxidative stress and NF-κB and MAPKs pathways activating. J Cell Biochem. (2018) 119:8981–95. doi: 10.1002/jcb.27154, 30076654

[113] Hu Q Zhang T Yi L Zhou X Mi M. Dihydromyricetin inhibits NLRP3 inflammasome-dependent pyroptosis by activating the Nrf2 signaling pathway in vascular endothelial cells. Biofactors. (2018) 44:123–36. doi: 10.1002/biof.1395, 29193391

[114] Schadich E Hlaváč J Volná T Varanasi L Hajdúch M Džubák P. Effects of ginger Phenylpropanoids and quercetin on Nrf2-ARE pathway in human BJ fibroblasts and HaCaT keratinocytes. Biomed Res Int. (2016) 2016:1–6. doi: 10.1155/2016/2173275, 26942188 PMC4749771

[115] Alharbi HOA Alshebremi M Babiker AY Rahmani AH. The role of quercetin, a flavonoid in the management of pathogenesis through regulation of oxidative stress, inflammation, and biological activities. Biomolecules. (2025) 15:151. doi: 10.3390/biom15010151, 39858545 PMC11763763

[116] Al-Khayri JM Sahana GR Nagella P Joseph BV Alessa FM Al-Mssallem MQ. Flavonoids as potential anti-inflammatory molecules: a review. Molecules. (2022) 27:2901. doi: 10.3390/molecules27092901, 35566252 PMC9100260

[117] Fang C Pan J Qu N Lei Y Han J Zhang J . The AMPK pathway in fatty liver disease. Front Physiol. (2022) 13:970292. doi: 10.3389/fphys.2022.970292, 36203933 PMC9531345

[118] Townsend LK Steinberg GR. AMPK and the endocrine control of metabolism. Endocr Rev. (2023) 44:910–33. doi: 10.1210/endrev/bnad012 37115289

[119] Xu Y Bai L Yang X Huang J Wang J Wu X . Recent advances in anti-inflammation via AMPK activation. Heliyon. (2024) 10:e33670. doi: 10.1016/j.heliyon.2024.e33670, 39040381 PMC11261115

[120] Inamdar S Joshi A Malik S Boppana R Ghaskadbi S. Vitexin alleviates non-alcoholic fatty liver disease by activating AMPK in high fat diet fed mice. Biochem Biophys Res Commun. (2019) 519:106–12. doi: 10.1016/j.bbrc.2019.08.139, 31472955

[121] von Loeffelholz C Coldewey SM Birkenfeld AL. A narrative review on the role of AMPK on de novo lipogenesis in non-alcoholic fatty liver disease: evidence from human studies. Cells. (2021) 10:1822. doi: 10.3390/cells10071822, 34359991 PMC8306246

[122] Jenkins Y Sun T-Q Markovtsov V Foretz M Li W Nguyen H . AMPK activation through mitochondrial regulation results in increased substrate oxidation and improved metabolic parameters in models of diabetes. PLoS One. (2013) 8:e81870. doi: 10.1371/journal.pone.0081870, 24339975 PMC3855387

[123] Wang Y An H Liu T Qin C Sesaki H Guo S . Metformin improves mitochondrial respiratory activity through activation of AMPK. Cell Rep. (2019) 29:1511–1523.e5. e5. doi: 10.1016/j.celrep.2019.09.070, 31693892 PMC6866677

[124] Hu P Li K Peng X Kan Y Li H Zhu Y . Nuclear receptor PPARα as a therapeutic target in diseases associated with lipid metabolism disorders. Nutrients. (2023) 15:4772. doi: 10.3390/nu15224772, 38004166 PMC10674366

[125] Małodobra-Mazur M Ołdakowska M Dobosz T. Exploring PPAR gamma and PPAR alpha’s regulation role in metabolism via epigenetics mechanism. Biomolecules. (2024) 14:1445. doi: 10.3390/biom14111445, 39595621 PMC11591816

[126] Singh S Kumar A Gupta S Agrawal R. Curative role of natural PPARγ agonist in non-alcoholic fatty liver disease (NAFLD). Tissue Barriers. (2024) 12:2289830. doi: 10.1080/21688370.2023.2289830, 38050958 PMC11262216

[127] Ngo J Choi DW Stanley IA Stiles L Molina AJA Chen PH . Mitochondrial morphology controls fatty acid utilization by changing CPT1 sensitivity to malonyl-CoA. EMBO J. (2023) 42:e111901. doi: 10.15252/embj.2022111901, 36917141 PMC10233380

[128] Niu X Han P Liu J Chen Z Zhang T Li B . Regulation of PPARγ/CPT-1 expression ameliorates cochlear hair cell injury by regulating cellular lipid metabolism and oxidative stress. Mol Gen Genomics. (2023) 298:473–83. doi: 10.1007/s00438-023-01993-8 36639590

[129] Wang Y Zhang M Liu J Li C Sun N Wu X . Novel therapeutic strategies for targeting fatty acid oxidation in cancer. Biomark Res. (2025) 13:145. doi: 10.1186/s40364-025-00855-2 41219902 PMC12607215

[130] Fan J Khanzada Z Xu Y. Mechanisms underlying muscle-related diseases and aging: insights into pathophysiology and therapeutic strategies. Muscles. (2025) 4:26. doi: 10.3390/muscles4030026, 40843913 PMC12371960

[131] Huynh C Ryu J Lee J Inoki A Inoki K. Nutrient-sensing mTORC1 and AMPK pathways in chronic kidney diseases. Nat Rev Nephrol. (2023) 19:102–22. doi: 10.1038/s41581-022-00648-y, 36434160

[132] Robert-Gostlin V. AMPK-Mediated mTORC1 Regulation in Macrophage Metabolism and Polarization. Ottawa, Canada: Université d'Ottawa/University of Ottawa (2024).

[133] Fan J Yuan Z Burley SK Libutti SK Zheng XFS. Amino acids control blood glucose levels through mTOR signaling. Eur J Cell Biol. (2022) 101:151240. doi: 10.1016/j.ejcb.2022.151240, 35623230 PMC10035058

[134] Zhao T Fan J Abu-Zaid A Burley SK Zheng XFS. Nuclear mTOR signaling orchestrates transcriptional programs underlying cellular growth and metabolism. Cells. (2024) 13:781. doi: 10.3390/cells13090781, 38727317 PMC11083943

[135] Fan J Zhang X Zhang J Zhao T Burley SK Zheng XFS. PDX1 phosphorylation at S61 by mTORC1 links nutrient signaling to β cell function and metabolic disease. Cell Rep. (2026) 45:116811. doi: 10.1016/j.celrep.2025.116811, 41528843 PMC12949489

[136] Kaldirim M Lang A Pfeiler S Fiegenbaum P Kelm M Bönner F . Modulation of mTOR signaling in cardiovascular disease to target acute and chronic inflammation. Front Cardiovasc Med. (2022) 9:907348. doi: 10.3389/fcvm.2022.907348, 35845058 PMC9280721

[137] Arner EN Rathmell JC. Metabolic programming and immune suppression in the tumor microenvironment. Cancer Cell. (2023) 41:421–33. doi: 10.1016/j.ccell.2023.01.009, 36801000 PMC10023409

[138] Smith TK Townsend LK Smiles WJ Oakhill JS Fullerton MD Steinberg GR. AMPK at the interface of nutrient sensing, metabolic flux and energy homeostasis. Nat Metab. (2026) 8:–51. doi: 10.1038/s42255-025-01442-3, 41526585

[139] Usui T Macleod MR McCann SK Senior AM Nakagawa S. Meta-analysis of variation suggests that embracing variability improves both replicability and generalizability in preclinical research. PLoS Biol. (2021) 19:e3001009. doi: 10.1371/journal.pbio.3001009, 34010281 PMC8168858

[140] Percie du Sert N Hurst V Ahluwalia A Alam S Avey MT Baker M . The ARRIVE guidelines 2.0: updated guidelines for reporting animal research. Br J Pharmacol. (2020) 177:3617–24. doi: 10.1111/bph.15193, 32662519 PMC7393194

[141] Hooijmans CR Rovers MM de Vries RB Leenaars M Ritskes-Hoitinga M Langendam MW. SYRCLE'S risk of bias tool for animal studies. BMC Med Res Methodol. (2014) 14:43. doi: 10.1186/1471-2288-14-43, 24667063 PMC4230647

[142] Li H Liang J Han M Gao Z. Polyphenols synergistic drugs to ameliorate non-alcoholic fatty liver disease via signal pathway and gut microbiota: a review. J Adv Res. (2025) 68:43–62. doi: 10.1016/j.jare.2024.03.004, 38471648 PMC11785558

[143] Liu Y-M Liu C Deng Y-S Chen Y Qiu Q-W Shang X-X . Beneficial effects of dietary herbs on high-fat diet-induced obesity linking with modulation of gut microbiota. Food Med Homol. (2025) 2:9420034. doi: 10.26599/FMH.2025.9420034

[144] Walsh SK Pettigrew K Mezzani I Alaswad I Bermano G. Role of selenium and 17β oestradiol in modulating lipid accumulation in in vitro models of obesity and NAFLD. Food Med Homol. (2025) 2:9420056. doi: 10.26599/FMH.2025.9420056

[145] Zhang J Chen Y Luo H Sun L Xu M Yu J . Recent update on the pharmacological effects and mechanisms of Dihydromyricetin. Front Pharmacol. (2018) 9:1204. doi: 10.3389/fphar.2018.01204, 30410442 PMC6209623

[146] Hou G Xu C Cheng K Mei S Kang Y Zhang C . Metabolic mechanisms of Dihydromyricetin and strategies for enhancing its bioavailability: a recent review. Food Chem. (2025) 485:144470. doi: 10.1016/j.foodchem.2025.144470, 40306054

