# Supplementary material for: Food-derived dihydromyricetin and metabolic dysfunction-associated steatotic liver disease: a preclinical systematic review and meta-analysis
Source: Front Nutr. 2026 Apr 7;13:1786758. doi: 10.3389/fnut.2026.1786758 (PMC13095842; doi:10.3389/fnut.2026.1786758)
Supplement: Supplementary file 1 [file Supplementary_file_1.docx]

***Supplementary Material***

1. **Supplementary Tables**

**Table S1. Characteristics of included studies**

| Author  (Year) | Nation | Sp.(strain) | Age  (Entry weight) | Gender | Study  design | N(I/C) | Modeling method | HFD composition  (source） | Methods of administration | DHM purity (source) | Dose (mg/kg) | Freq | T(w) | Outcome measures |
| --- | --- | --- | --- | --- | --- | --- | --- | --- | --- | --- | --- | --- | --- | --- |
| ChengJ  (2024) | China | Mice  (C57BL/6J) | 7w  (18-20g) | M | RAS | 10/10 | HFD | 60% kcal from fat diet (research diets, D12492, USA) | oral | Purity not reported (Derick Biotechnology Co., Ltd. ,Chengdu, China) | 300 | In diet daily | 11 | BW, serum TG,Tche,LDL,ALT,AST, hepatic MDA,SOD,GSH,GSH-Px,CAT, IL-6, IL-1β |
| KangL  (2024) | China | Mice  (C57BL/6J) | 3-4w  (NA) | M | RAS | 10/10 | HFD | 60% kcal from fat (TestDiet, 58Y1, MO, USA) | oral gavage | 98% (Shanghai Biotechnology Co., Ltd., China) | 1000 | QD | 8 | BW,liver index, serum TG,Tche, LDL, HDL, ALT, AST, ALP, TNF-α |
| LengQY  (2022) | China | Mice  (C57BL/6J) | 6-8w  (NA) | M | RAS | 6/6 | HFD | 60% kcal from saturated fat (Research Diets, New Brunswick, NJ,USA) | oral gavage | Purity not reported (GuangRun Bio Technology Co., Ltd. , Nanjing, China) | 100 | QD | 4 | BW, serum TG, Tche |
| LiJ  (2024) | China | Mice  (C57BL/6J) | 8w  (NA) | M | RAS | 8/8 | HFD | Composition and source not reported | oral gavage | >99% (prepared in-house, source not reported) | 25 | QD | 8 | BW, liver index, serum TG, Tche, LDL, HDL, ALT, AST, ALP, hepatic MDA, SOD, GSH, |
| LiuL  (2023) | China | LDLR^-/^ mice  (C57BL/6J) | 4w  (NA) | M | RAS | 8/8 | HFD | 20% kcal from fat, 0.5% kcal from cholesterol, and 35% kcal from fructose (source not reported) | oral gavage | 98% (Jiangsu Enming Bioengineering Technology Co. Ltd., China) | 250 | QD | 8 | BW, liver index, serum TG, LDL, HDL, FBG, insulin, ALT, AST, hepatic TG |
| LvH  (2022) | China | Mice  (C57BL/6J) | 3-4w  (16-19g) | M | RAS | 6/6 | HFD | Composition and source not reported | oral gavage | Purity and source not reported | 250 | QD | 16 | BW, serum TG, Tche, LDL, HDL, hepatic MDA, SOD, TNF-α, IL-6, CPT-1, PPARα |
| LyuQ  (2022) | China | Mice  (ICR) | 4w  (15-18g) | M | RAS | 6/6 | HFD | 78.8% basic feed + 10% lard + 10% whole egg yolk powder + 1.0% cholesterol + 0.2% bile salt (prepared in-house, source not reported) | oral gavage | 98.0% (prepared in-house, source not reported) | 100 | QD | 5 | BW, serum TG, Tche, LDL, HDL, hepatic TG, Tche |
| ZengX  (2019) | China | Mice  (C57BL/6J) | 8w  (NA) | M | RAS | 5/6 | HFD | 45% kcal from fat  (Research Diets, NJ, USA) | oral gavage | ≥98% (Chengdu Mansite Bio-Technology Co., Ltd., Chengdu, China) | 300 | QD | 12 | BW, liver index, serum TG, Tche, LDL, HDL, ALT, hepatic TG, MDA, pAMPK/AMPK ratio |
| ZengY  (2020) | China | LDLR ^−/−^ mice (C57BL/6JNju) | 48w  (NA) | M | RAS | 6/6 | HFD | 21% kcal from fat and 0.21% kcal from cholesterol ( Open Source Diets, Research Diets, Inc, Changzhou, China) | oral gavage | purity > 98%, (Xi’an Natural Field Biotechnioue Co., LTD, Xi’an, China) | 400 | QD | 12 | BW, serum TG, Tche, LDL, HDL, FBG, insulin, ALT, AST, hepatic MDA, SOD, GSH-Px, CAT, TNF-α, IL-6, SIRT1, PPARα, pAMPK/APMK ratio |
| RanL  (2024) | China | Mice  (C57BL/6J) | 8w  (21-24g) | M | RAS | 8/8 | HFD | XTMRCD60; 60% kcal from fat, 0.1% kcal from methionine; choline-deficient (Jiangsu Xietong Medical Bioengineering Co., Ltd., China) | oral gavage | ≥99% (Chengdu Mansite Bio-Technology Co., Ltd., China) | 200 | QD | 4 | BW, liver index, serum ALT, AST, hepatic TG, Tche,PPAR-α, SOD, GSH, TNF-α, IL-1β |
| MaX  (2018) | China | Mice  (C57BL/6J) | 6w  (18.7-20.7g) | M | RAS | 15/15 | HFD | 35% kcal from carbohydrate, 20% kcal protein; 45% kcal from fat (Beijing HFK Bioscience H10045, Beijing, China) | oral gavage | >98％(Chengdu Mansite Bio-Technology Co., Ltd., China) | 100 | QD | 12 | BW, liver index, serum TG, Tche, LDL, HDL, FBG, insulin, |
| JiangL  (2023) | China | Mice  (C57BL/6J) | 6w(NA) | M | RAS | 10/10 | HFD | 35% kcal from carbohydrate, 20% kcal from protein; 45% kcal from fat (Beijing HFK Bioscience H10045, Beijing, China) | oral gavage | Purity not reported (Chengdu Mansite Bio-Technology ,China) | 300 | QD | 12 | BW, liver index, serum TG, Tche, LDL, HDL, FBG, ALT, AST, |
| LengQ  (2020) | China | Mice  (C57BL/6J) | 7w(NA) | M | RAS | 15/15 | HFD | 60% kcal from fat (research diets, USA) | oral gavage | Purity not reported (GuangRun Bio Technology Co., Ltd. , Nanjing, China) | 100 | QD | 4 | BW, Liver index, serum TG, Tche, FBG, insulin, ALT, AST, hepatic TG, PPAR-α, CPT-1, TNF-α, IL-1β, IL-6 |
| LiuH  (2017) | China | ApoE^−/−^ mice  (C57BL/6J) | 8w  (18-22g) | M | RAS | 10/10 | HFD | 20% kcal from fat, 0.3% kcal from cholesterol (Beijing HFK Bioscience H10045, Beijing, China) | oral gavage | >98％, (ShanghaiRonghe Pharmaceutical Technology Development Co., Ltd., Chna) | 100 | QD | 12 | Serum TG, Tche, LDL, HDL, ALT, AST, hepatic SOD, CAT, MDA |

Abbreviations: DHM, dihydromyricetin; RAS, randomized animal study; QD, once daily; HFD, high-fat diet; BW, body weight; TG, triglycerides; LDL, low-density lipoprotein; HDL, high-density lipoprotein; ALT, alanine aminotransferase; AST, aspartate aminotransferase; MDA, malondialdehyde; SOD, superoxide dismutase; GSH, glutathione; GSH-Px, glutathione peroxidase; CAT, catalase; TNF, tunor necrosis factor; IL, interleukin; FBG, fasting blood glucose; M, male; N, number; NA, not available; I/C, intervention/control; T, treatment; w, weeks; Freq, frequency.

**T****able S2. Results of Egger’s test for publication bias**

| group | variable | Egger's p value |
| --- | --- | --- |
| liver injury | ALT | 0.45 |
| anthropometric | BW | 0.25 |
| serum lipid | serum TG | 0.60 |
|  | serum Tche | 0.01 |
|  | serum LDL | 0.09 |
|  | serum HDL | 0.09 |

Abbreviations: ALT, alanine aminotransferase; BW, body weight; TG, triglycerides; LDL, low-density lipoprotein; HDL, high-density lipoprotein.

1. **Supplementary Figures**


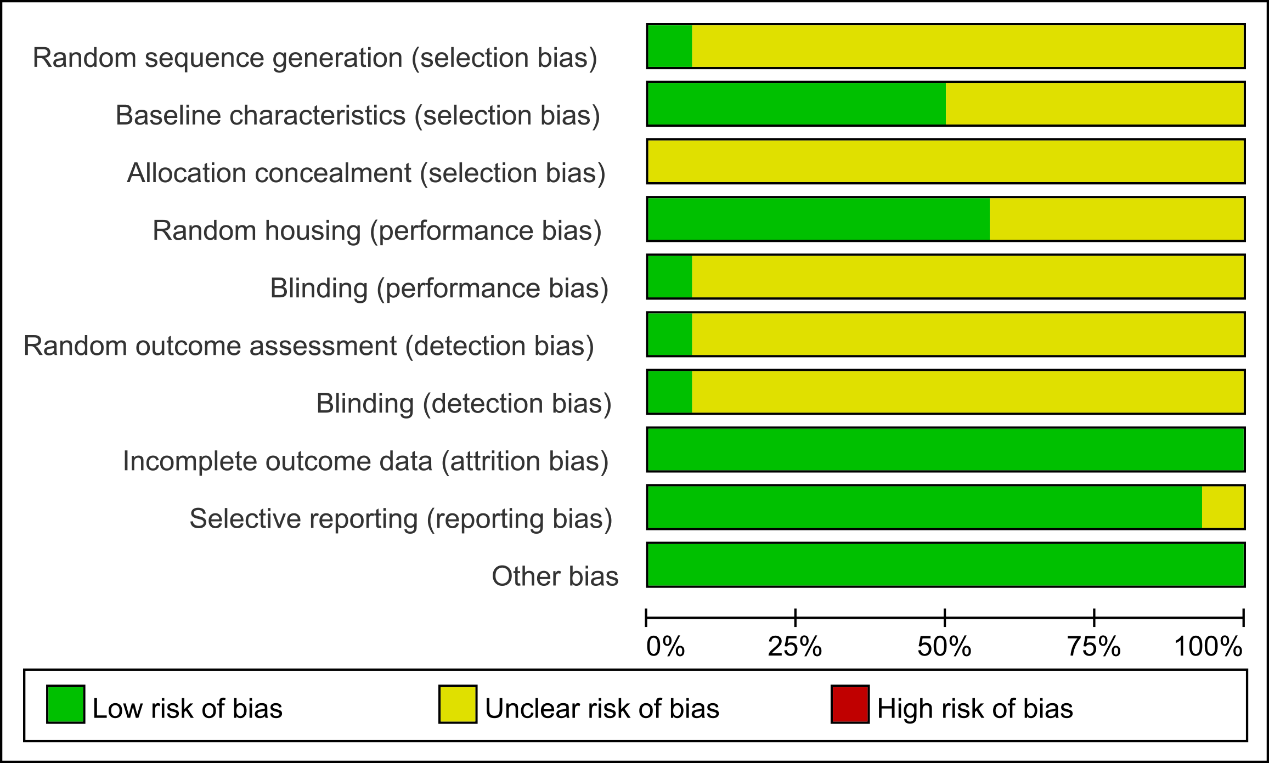


**Supplementary Figure S1** **Risk of bias graph: review authors’ judgments about each risk of bias item presented as percentages across all included studies.**


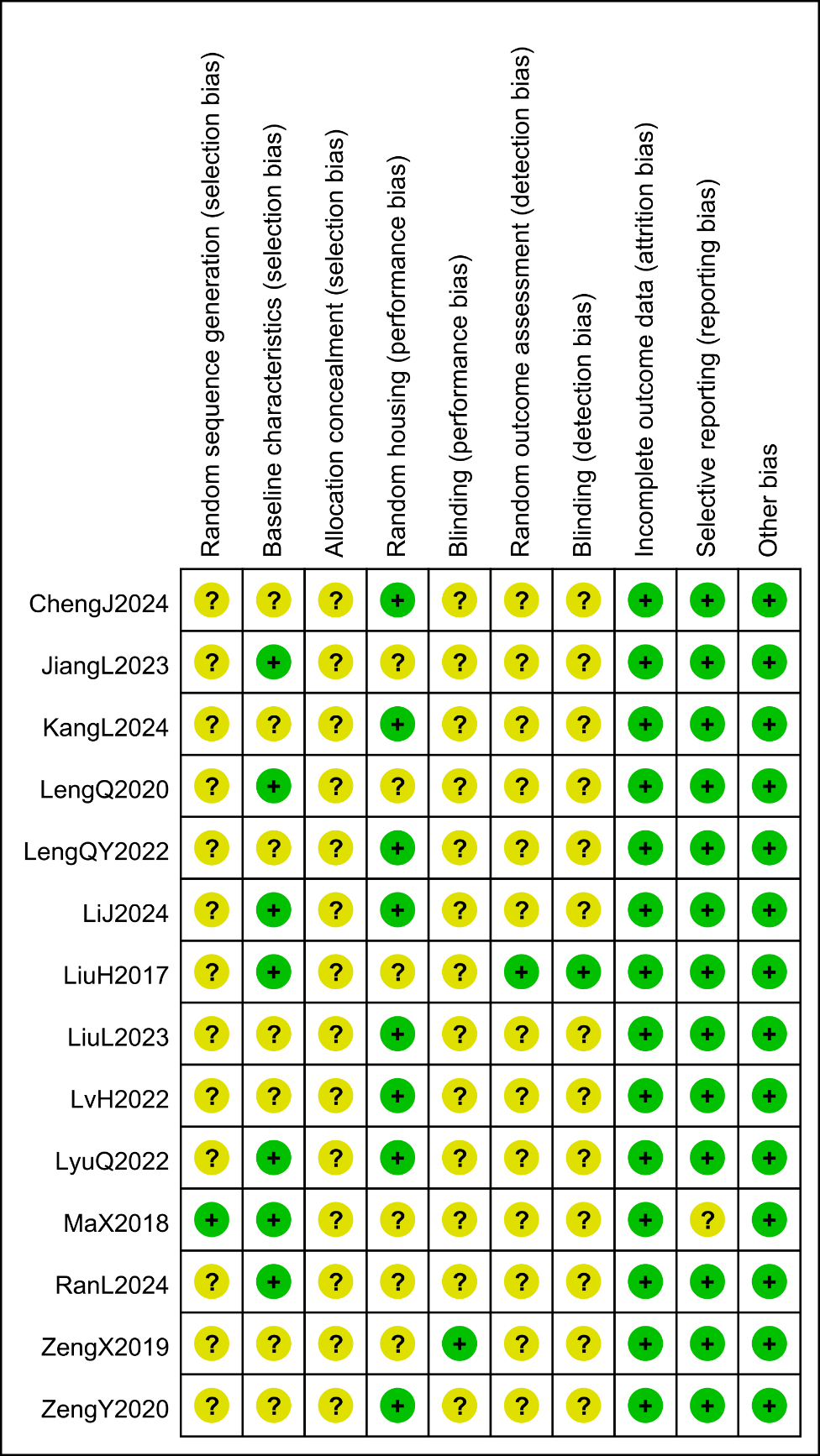


**Supplementary Figure S2 Risk of bias summary: review authors’ judgements about each risk of bias item for each included study**


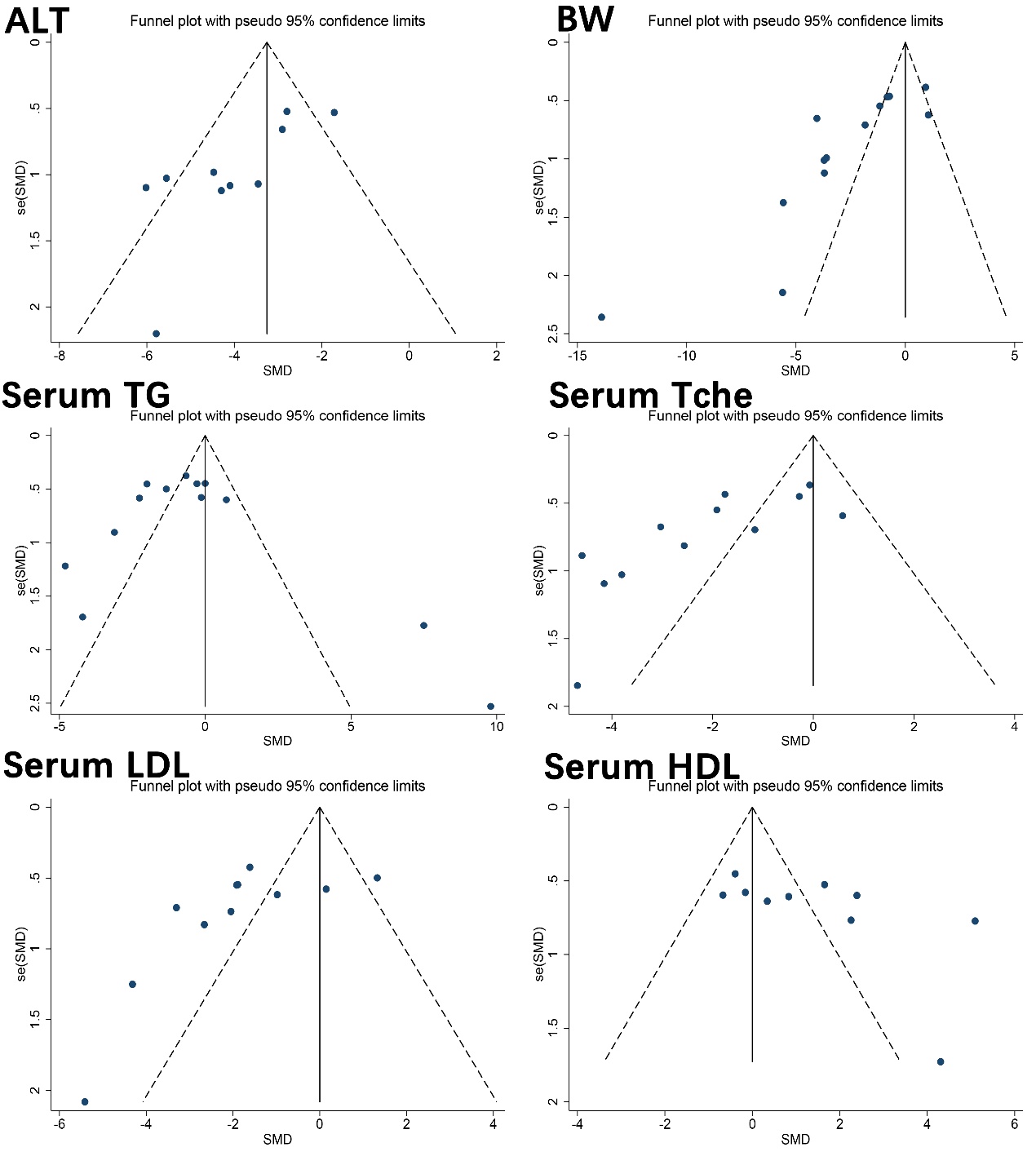


**Supplementary Figure S3** **Funnel plots assessing publication bias for outcomes with more than 10 studies (ALT, BW, serum TG, Tche, LDL, and HDL)**. ALT, alanine aminotransferase; BW, body weight; TG, triglyceride; Tche, total cholesterol; LDL, low-density lipoprotein cholesterol; HDL, high-density lipoprotein cholesterol.


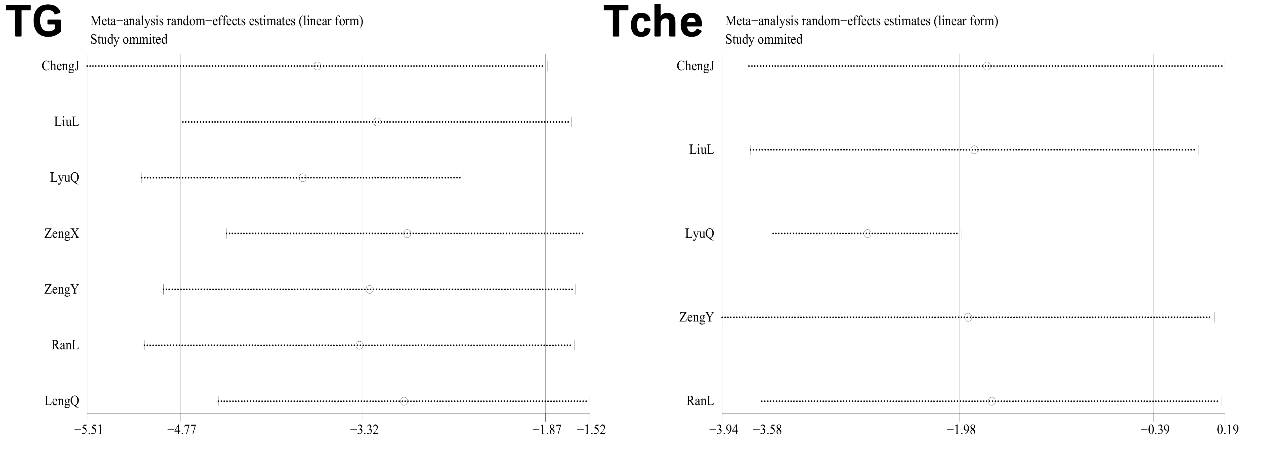
**Supplementary Figure S4** **Sensitivity analysis of the effect of DHM on hepatic lipid profiles.** DHM, dihydromyricetin; TG, triglyceride; Tche, total cholesterol.


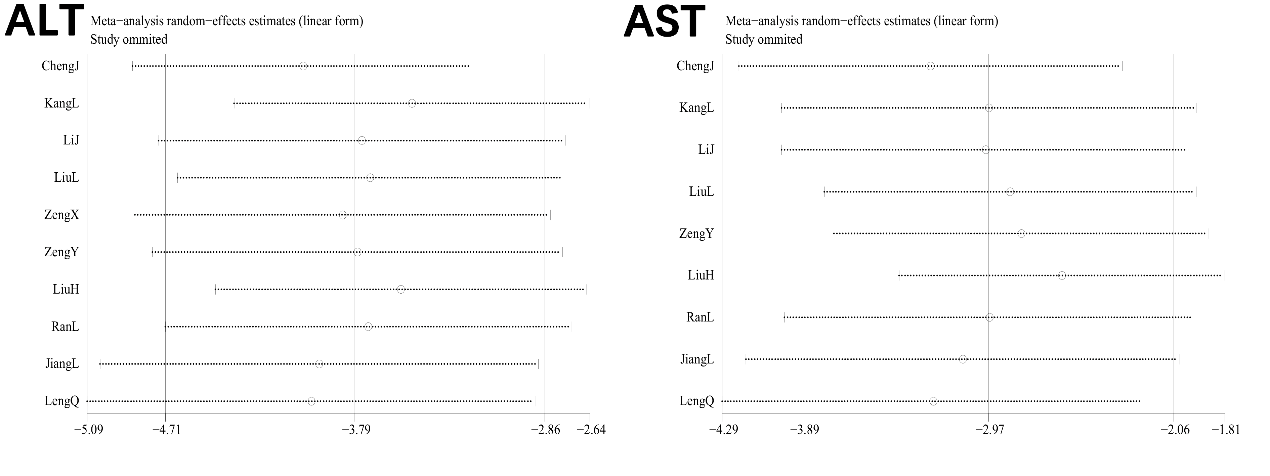
**Supplementary Figure S5** **Sensitivity analysis of the effect of DHM on liver enzymes.** DHM, dihydromyricetin; ALT, alanine aminotransferase; AST, aspartate aminotransferase.


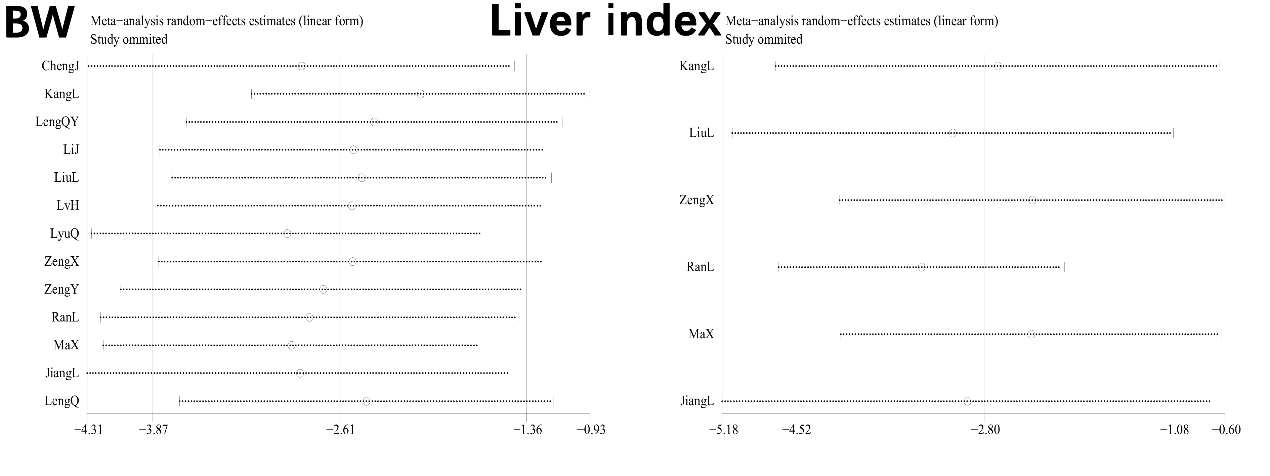
**Supplementary Figure S6** **Sensitivity analysis of the effect of DHM on anthropometric parameters.** DHM, dihydromyricetin; BW, body weight.

**
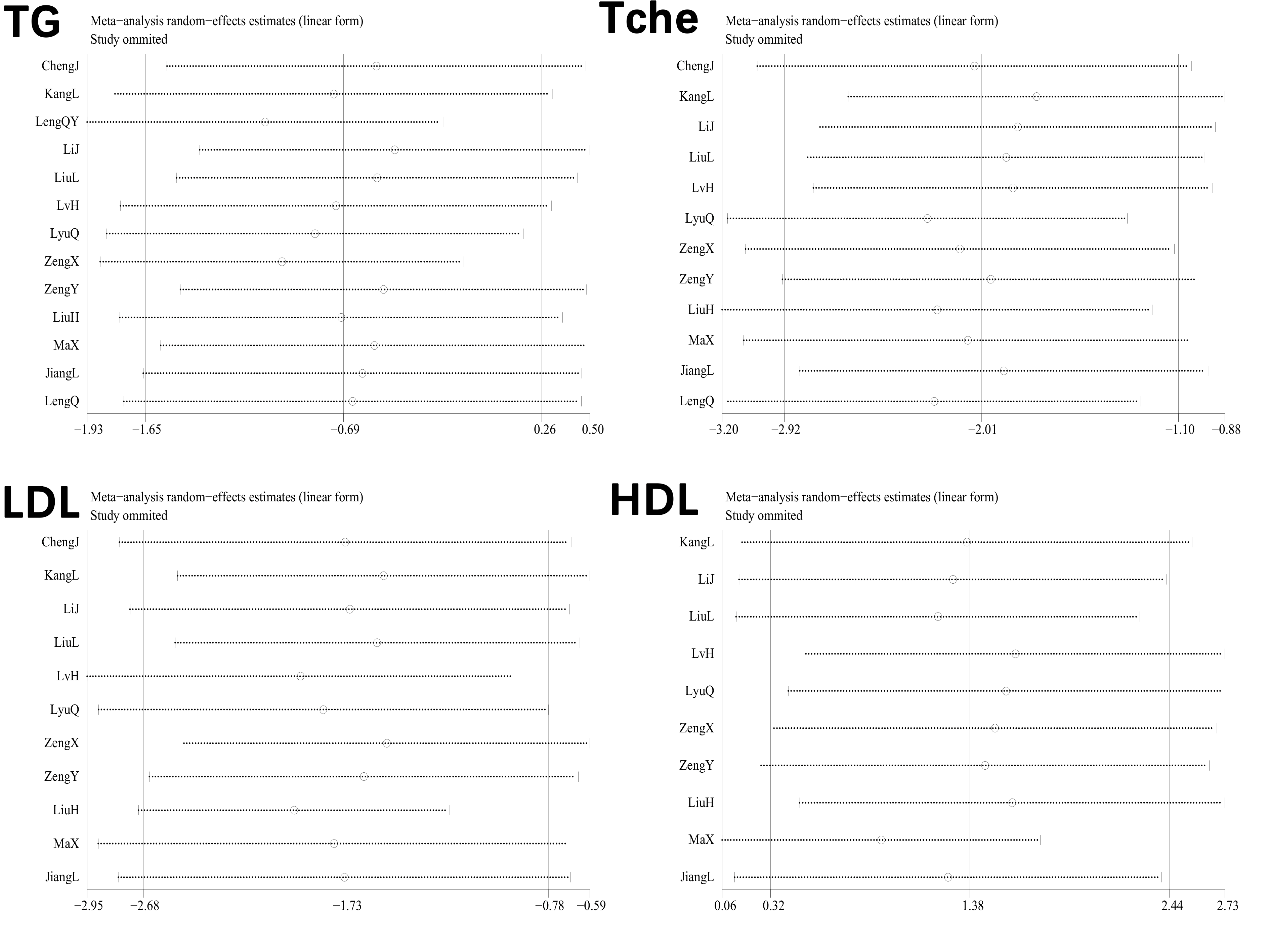
**

**Supplementary Figure S7** **Sensitivity analysis of the effect of DHM on serum lipid profiles.** DHM, dihydromyricetin; TG, triglyceride; Tche, total cholesterol; LDL, low density lipoprotein cholesterol; HDL, high density lipoprotein cholesterol.

**
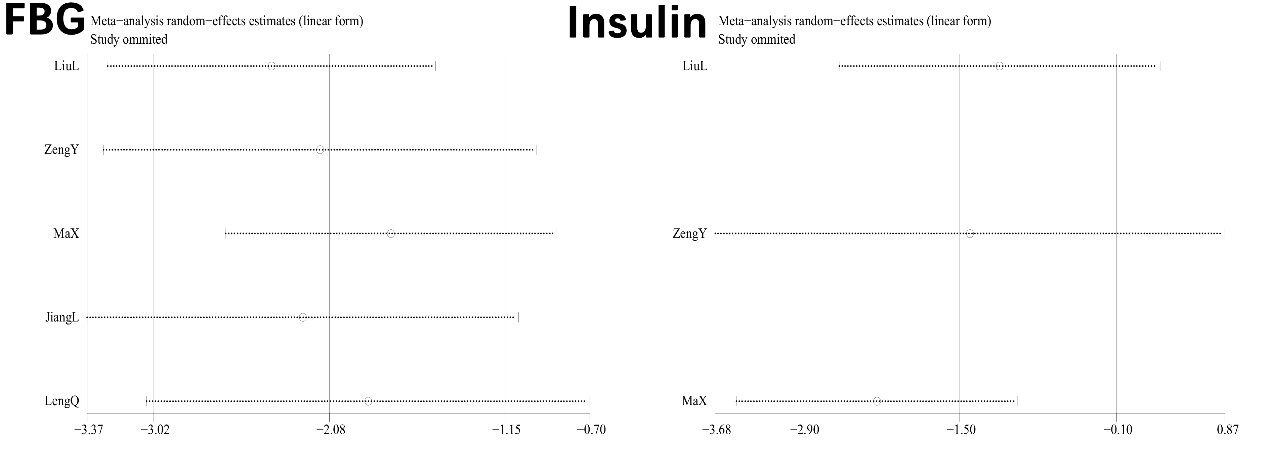
**

**Supplementary Figure S8** **Sensitivity analysis of the effect of DHM on glucose metabolism.** DHM, dihydromyricetin; FBG, fasting blood glucose.

**
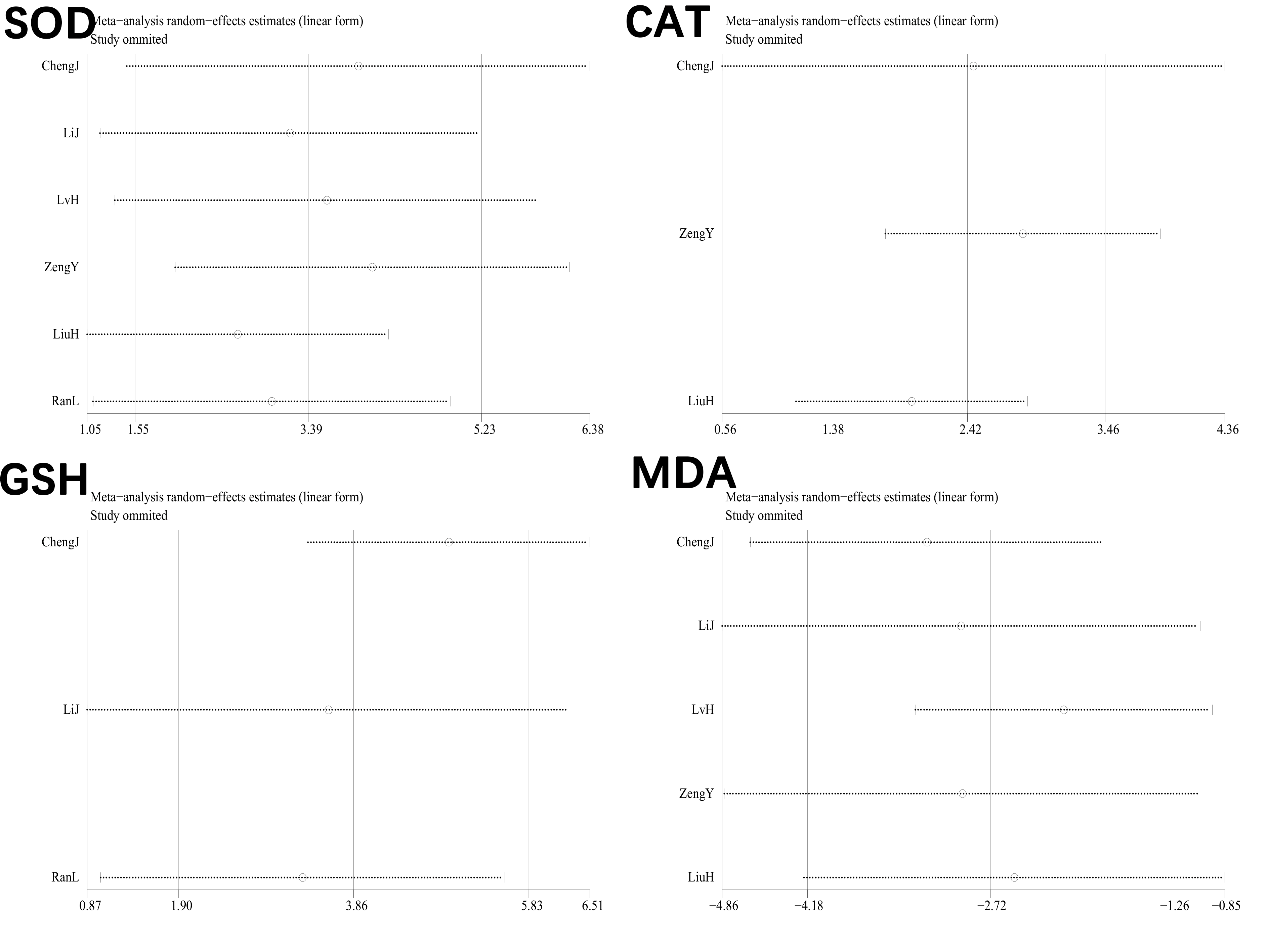
**

**Supplementary Figure S9 Sensitivity analysis of the effect of DHM on oxidative stress markers.** DHM, dihydromyricetin; MDA, malondialdehyde; SOD, superoxide dismutase; GSH, glutathione; CAT, catalase.

**
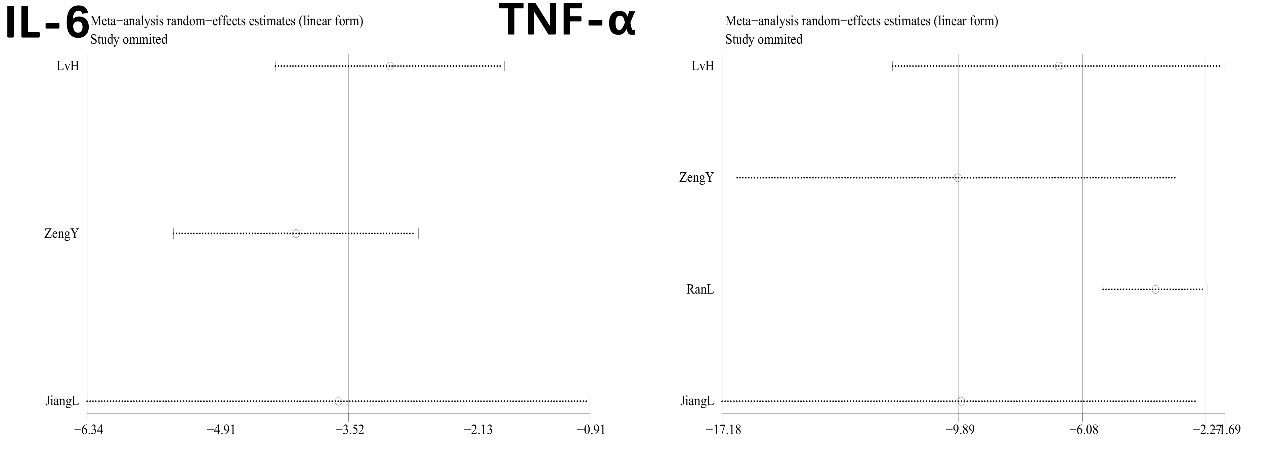
Supplementary Figure S10** **Sensitivity analysis of the effect of DHM on inflammatory cytokines.** DHM, dihydromyricetin; TNF, tumor necrosis factor ; IL, interleukin.

**
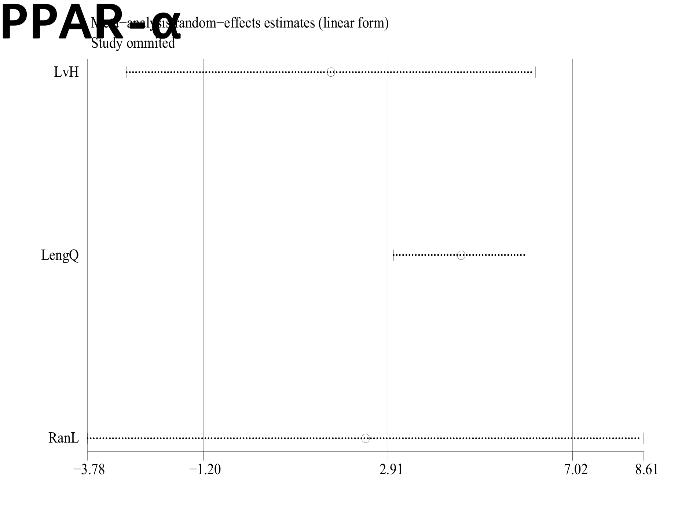
**

**Supplementary Figure S11** **Sensitivity analysis of the effect of DHM on PPAR-α (the only signaling protein outcome reported in ≥ 3studies).** PPAR-α, peroxisome proliferator-activated receptor-α.

**
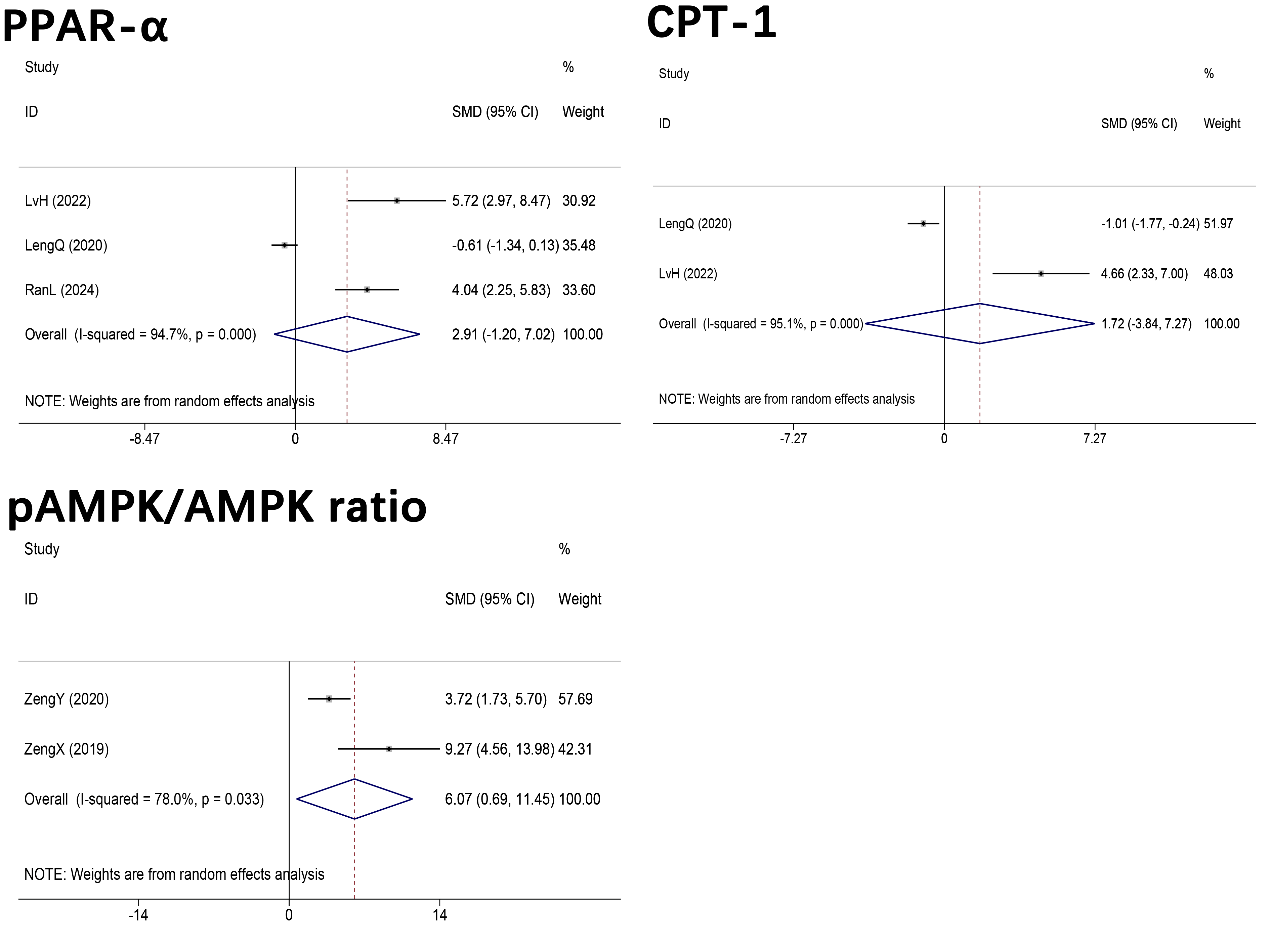
Supplementary Figure S12 Forest plot for the effect of DHM on hepatic signaling proteins.** DHM, dihydromyricetin. PPAR-α, peroxisome proliferator-activated receptor-α; CPT-1, carnitine palmitoyltransferase-1; pAMPK, phosphorylated AMP-activated protein kinase; AMPK, AMP-activated protein kinase.

**
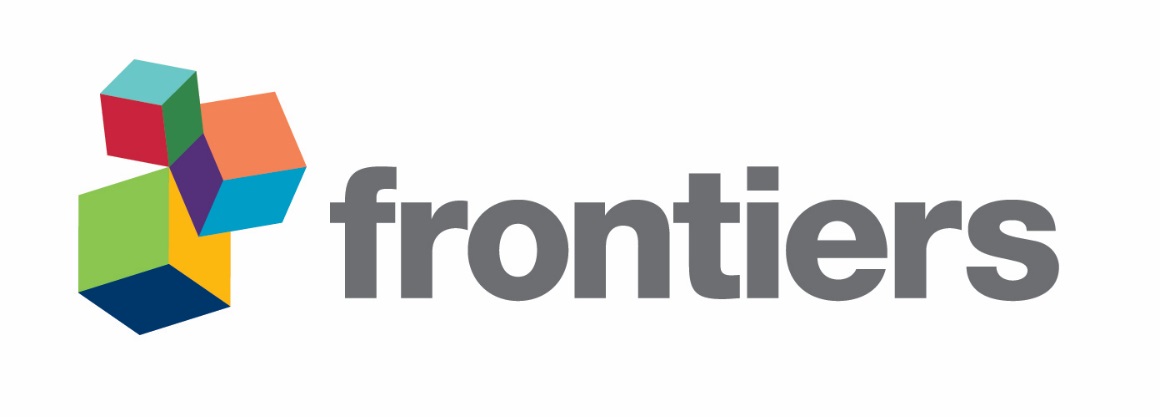
**
